# First principles study of dense and metallic nitric sulfur hydrides

**DOI:** 10.1038/s42004-021-00517-y

**Published:** 2021-06-04

**Authors:** Xiaofeng Li, Angus Lowe, Lewis Conway, Maosheng Miao, Andreas Hermann

**Affiliations:** 1 College of Physics and Electronic Information, Luoyang Normal University, Luoyang, China; 2 Centre for Science at Extreme Conditions and SUPA, School of Physics and Astronomy, The University of Edinburgh, Edinburgh, UK; 3 Department of Chemistry & Biochemistry, California State University, Northridge, CA USA; 4 Department of Earth Science, University of California Santa Barbara, CA, USA

**Keywords:** Density functional theory, Structure prediction, Structure of solids and liquids, Electronic properties and materials

## Abstract

Studies of molecular mixtures containing hydrogen sulfide (H_2_S) could open up new routes towards hydrogen-rich high-temperature superconductors under pressure. H_2_S and ammonia (NH_3_) form hydrogen-bonded molecular mixtures at ambient conditions, but their phase behavior and propensity towards mixing under pressure is not well understood. Here, we show stable phases in the H_2_S–NH_3_ system under extreme pressure conditions to 4 Mbar from first-principles crystal structure prediction methods. We identify four stable compositions, two of which, (H_2_S) (NH_3_) and (H_2_S) (NH_3_)_4_, are stable in a sequence of structures to the Mbar regime. A re-entrant stabilization of (H_2_S) (NH_3_)_4_ above 300 GPa is driven by a marked reversal of sulfur-hydrogen chemistry. Several stable phases exhibit metallic character. Electron–phonon coupling calculations predict superconducting temperatures up to 50 K, in the *Cmma* phase of (H_2_S) (NH_3_) at 150 GPa. The present findings shed light on how sulfur hydride bonding and superconductivity are affected in molecular mixtures. They also suggest a reservoir for hydrogen sulfide in the upper mantle regions of icy planets in a potentially metallic mixture, which could have implications for their magnetic field formation.

## Abstract (web summary)

Hydrogen sulfide and ammonia form molecular mixtures that could open up new routes towards hydrogen-rich high-pressure/high-temperature superconductors, but their mixing propensity under pressure is not well understood. Here, the authors identify stable nitric sulfur hydrides at high pressure using first-principles crystal structure prediction methods.

## Introduction

Dense molecular systems are of significant interest to different scientific fields. The high-pressure properties of hydrogen sulfide (H_2_S) have been of tremendous interest to condensed matter, solid state chemistry and materials science research, due to the conventional superconductivity found in H_2_S above 200 K at pressures around 155 GPa^1–6^. Recent predictions of a superconducting metastable H_3_S–CH_4_ compound further highlight the materials science aspect of studying dense molecular mixtures^7,8^ and a carbonaceous sulfur hydride compound has been reported as the first room-temperature superconductor^9^. However, the composition or structure of the superconducting material are unknown. To develop a detailed understanding of what drives the superconductivity, with the ultimate aim of reducing the pressures required to generate the superconducting state, systematic investigations of the interaction of H_2_S with molecular species at high pressures are needed, but these are mostly missing.

A secondary interest in dense molecular mixtures comes from planetary science. The mantle regions of the icy giants of our solar system are dominated by “hot ice” layers that consist of mixtures dominated by water, methane and ammonia at extreme pressure-temperature conditions up to several Mbar and several thousand Kelvin^10–12^. Unlike the Jovian planets, which are dominated throughout by hydrogen/helium, complex chemical processes are expected for “hot ice” mixtures of molecular fluids at deep planetary conditions^13,14^, including losing their original molecular nature, the formation of exotic states such as “superionic” water or ammonia, potential demixing, etc^15,16^. The physical and chemical properties of the “hot ice” layers greatly influence the gravitational moments, rotational velocities and atmospheric composition as well as the thermal evolution and internal structure of these celestial bodies. Realistic studies of the “hot ice” mixtures are paramount to expand our understanding of these planetary environments. High-pressure experiments, e.g., on “synthetic Uranus”, hydrocarbons, and binary molecular mixtures have shown diverse chemistry and unexpected reactivity under specific pressure-temperature conditions^17–19^. Meanwhile, electronic structure calculations have studied various mixtures of molecular ices and their interactions with the lighter atmosphere constituents hydrogen and helium and predict a plethora of stable compounds and exotic states of matter, such as plasticity and staged superionicity^20–27^. H_2_S, despite confirmed via atmospheric observations of Uranus and Neptune to exist in those planets^28–30^, is considered a minor component of their overall composition and its mixtures are little studied.

A molecular compound that will react with H_2_S is ammonia, NH_3_. In a planetary context, this mixture is relevant as ammonium hydrosulfide (NH_4_SH) and ammonium sulfide ((NH_4_)_2_S) are the major H_2_S reservoirs in clouds in the lower atmospheres of Uranus, Neptune, and the Jovian planets^31,32^. However, the atmospheric abundances of nitrogen and sulfur inferred from microwave absorption experiments are heavily distorted from solar ratios, and constraints of interior S/N and N/H ratios are complicated by possible sequestration of H_2_S or NH_3_ deeper inside the planets’ icy oceans^32–35^. While NH_4_SH has been known since the 19^th^ century^36^ and its crystal structure and infrared (IR) and optical absorption properties have been studied extensively^37–41^, there are no studies of high-pressure mixtures of H_2_S and NH_3_. Computational studies of the analog water–ammonia system predicted the formation of ionic compounds at high pressure, stabilized in part by full disintegration of the water molecule^21,42,43^, which has recently been confirmed by experiment^44^. If the analogy to water–ammonia mixtures holds for compressed H_2_S-NH_3_, one may expect novel compositions under pressure and formation of ionic compounds, but in addition also metallic phases that could lead to new insights into hydride superconductors.

There are therefore multiple motivations to study molecular mixtures that include H_2_S at extreme conditions: (i) Does the pressure-induced metallization and superconductivity of H_2_S and H_2_S–CH_4_ mixtures translate to other relevant molecular mixtures? (ii) Can analogies be drawn to the behavior of hydrogen sulfide’s homolog, water, with its predicted de-protonated ionic phases in mixtures with ammonia? (iii) Can the observation of hydrogen sulfide in icy planets’ atmospheres be explained by a reservoir of stable compounds at elevated pressures? Understanding mixtures of H_2_S and NH_3_ under compression not only has important consequences for materials science and the chemistry of dense hydrogen-bonded molecular compounds but also for expanding our horizon of complex materials in planetary interiors.

We study here the formation of nitric sulfur hydrides in the form of hydrogen sulfide-ammonia mixtures under high-pressure conditions up to 400 GPa using crystal structure prediction and ab-initio calculations, and present a theoretical overview of their phase diagrams, including stable metallic and superconducting phases with novel bonding configurations, driven by a reversal of sulfur-hydrogen chemistry that transforms sulfur from an electronegative isolated S^2−^ anion to an electropositive S^6+^ polyhedra-former.

## Results

### Stable Compounds

**Fig. 1:**
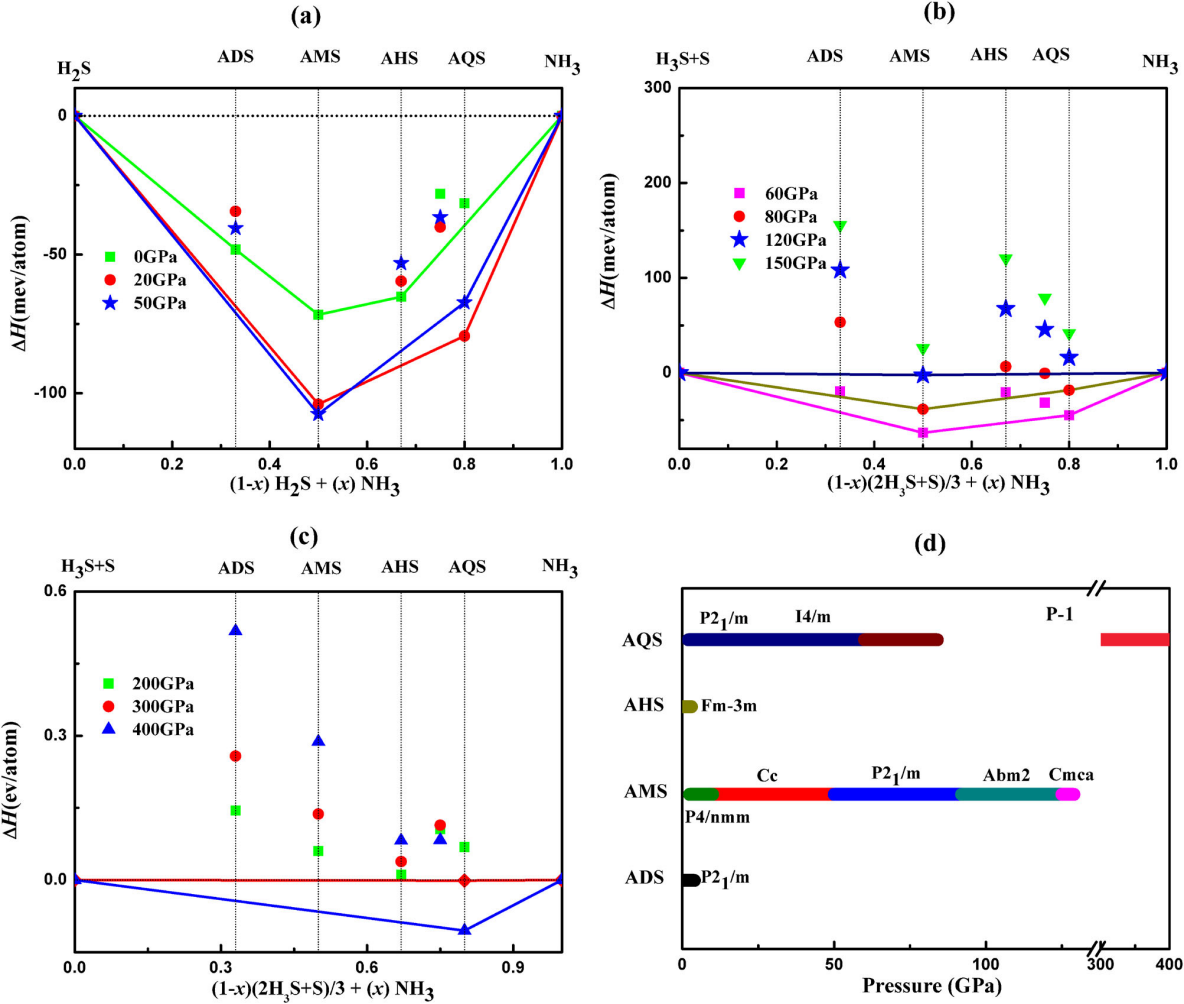
Convex hulls and ground state phase diagram of the H_2_S-NH_3_ system. **a** Convex hulls relative to H_2_S and NH_3_ up to 50 GPa, **b** convex hulls relative to H_3_S + S and NH_3_ from 60 to 150 GPa, **c** convex hulls relative to H_3_S + S and NH_3_ from 200 to 400 GPa, and **d** ground state phase diagram under pressure. Colored bars indicate stability ranges of phases as labeled by space group, for different H_2_S-NH_3_ mixtures.

To find the stable H_2_S-NH_3_ compounds, we performed extensive structure searches on various compositions of (H_2_S)_*x*_ (NH_3_)_*y*_ under pressure. Amongst potential mixtures we highlight the compositions ammonia mono-sulfide (AMS, NH_3_:H_2_S = 1:1), ammonia di-sulfide (ADS, 1:2), ammonia hemi-sulfide (AHS, 2:1), and ammonia quarter-sulfide (AQS, 4:1). Figure 1a exhibits the resulting convex hulls of the binary compounds of H_2_S-NH_3_ below 60 GPa. At zero pressure, we find three stable compounds, AMS, ADS, and AHS. Both ADS and AHS become unstable relatively quickly, at 5 GPa and 3 GPa, respectively (Supplementary Figs. 6–9 in the Supplementary Discussion show detailed enthalpy plots). Instead, when pressure increases above 2 GPa, the most ammonia-rich compound AQS becomes stable. The convex hull above 60 GPa (relative to ammonia, H_3_S and S) is shown in Fig.1b, c. AQS remains stable up to 83 GPa, above which it decomposes into AMS + NH_3_. AMS remains stable up to 129 GPa before decomposing into the constituent ices NH_3_, H_3_S and S. Among all compounds, AMS is the most thermodynamically stable mixture throughout the low pressure range, i.e., has the largest formation enthalpy per molecule. What is particularly interesting is the re-entrant stability of AQS above 300 GPa (Fig. 1c, Supplementary Fig. 8), which is due to intriguing changes in chemistry we will examine further below.

The ground state stability ranges for all compounds are summarized in the phase diagram Fig. 1d, which also outlines all phase transitions as function of pressure. Zero-point vibrational energies can play an important role in influencing the phase stabilities of compounds containing light elements such as hydrogen^22^. We therefore examined the effect of zero-point energies on the stability of the stoichiometries on the convex hull, see Supplementary Fig. 10. Inclusion of ZPE leads to lowering of all formation enthalpies, making mixtures more stable with respect to their constituents, but does not change the qualitative picture of stabilization and decomposition in various pressure regimes. Another avenue to consider is that, above 50 GPa, with both H_3_S and S available from decomposition of H_2_S, ternary mixtures of composition NH_3_–H_3_S–S could become stable. Exploratory searches around simple H_3_S:S mixing ratios of 1:1, 2:1, and 1:2 did not find phases more stable than mixtures involving AMS, see Supplementary Fig. 11. All these molecular mixtures are part of the N–S–H ternary, a full study of which is beyond the scope of this work. Properties of the metastable ATS phases are presented in the Supplementary Discussion and Supplementary Figs. 12–15.

### Structures and phase transitions

**Fig. 2:**
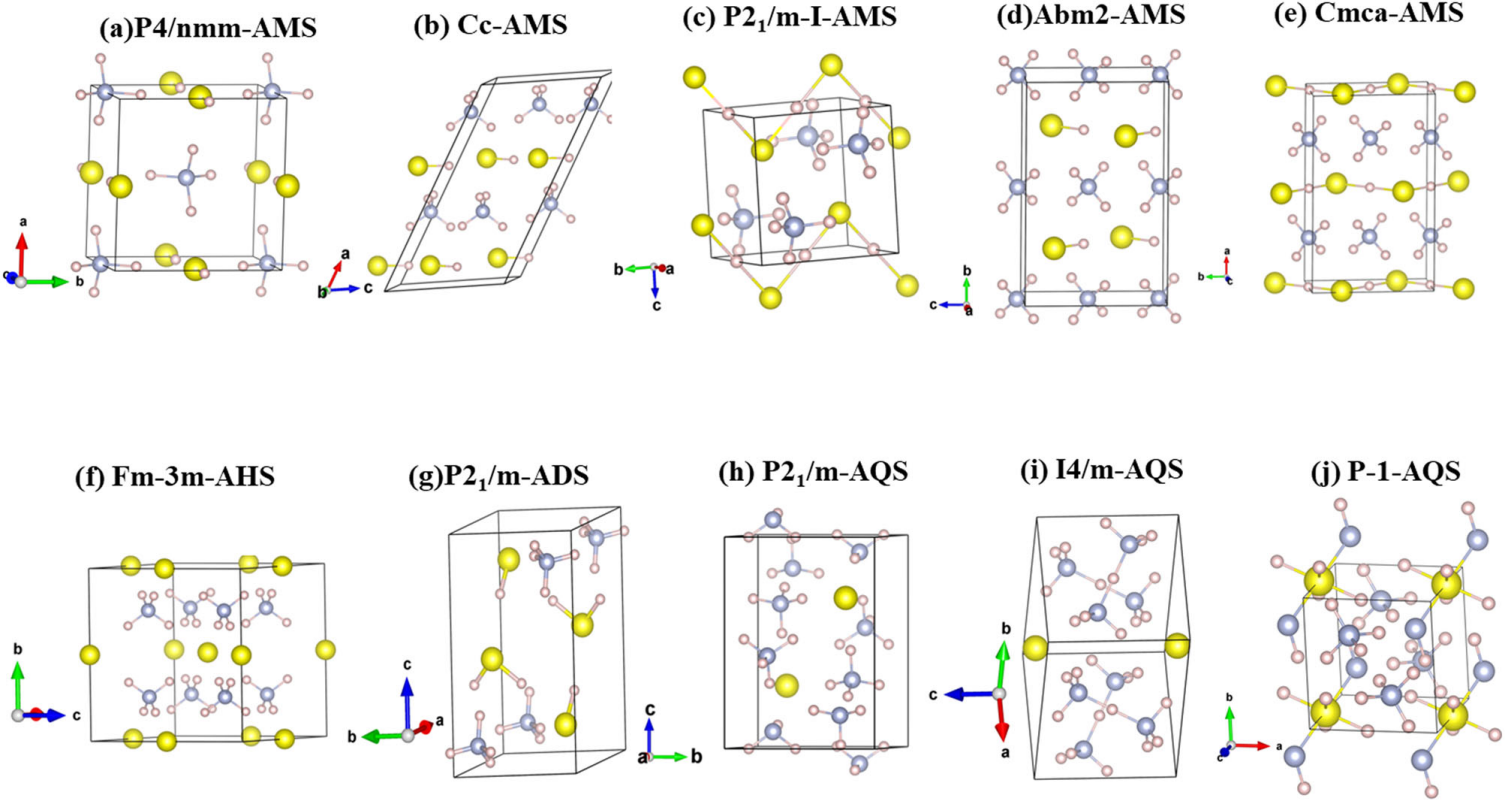
Crystal structures of stable compounds in the H_2_S-NH_3_ system. **a**–**j** Crystal structures of H_2_S-NH_3_ structures as labeled. Yellow (blue, pink) spheres denote sulfur (nitrogen, hydrogen) atoms, and covalent bonds are indicated by thin lines. Unit cells and crystallographic axes are as shown.

For AMS at ambient conditions, we successfully reproduce the low-pressure tetragonal *P4/nmm* phase as reported by Bragin et al.^37^. This is the same structure adopted by AMH (NH_3_:H_2_O) at pressures above 10 GPa^21^. We find that AMS should transform from *P4/nmm* to a monoclinic *Cc* phase and further to a *P2*_*1*_*/m* phase at 11 GPa and 50 GPa respectively, followed by orthorhombic *Abm2* at 94 GPa and *Cmma* at 125 GPa. The low pressure *P4/nmm* and *Cc* phases, shown in Fig. 2a, b, form simple ionic (NH_4_)^+^(SH)^−^ arrangements; they differ in the layout of the S–H…S–H hydrogen bonded chains, which are linear in *P4/nmm* and kinked in *Cc*. *P2*_*1*_*/m* (Fig. 2c) represents an evolution of the *Cc* phase, in which (SH)^−^ anions form hydrogen-bonded 1D chains with linear symmetric S–H–S bonds; the pressure for hydrogen bond symmetrization (50 GPa) is consistent with that seen in other ices and minerals^45,46^. At higher pressure, the orthorhombic phase *Abm2* (Fig. 2d) returns to the ionic (NH_4_)^+^(SH)^−^ motif while *Cmma* (Fig. 2e) has kinked infinite –(H–S)- chains with symmetric buckled S–H-S bonds in a matrix of NH_4_^+^ cations; structural motifs seen for instance throughout the alkali hydroxides M^+^(OH^−^)^47^.

For ammonium sulfide, (NH_4_)_2_S, here labeled AHS, we find a high-symmetry *Fm-3m* phase (Fig. 2f) as the stable ground state at zero pressure. It is an anti-fluorite structure with NH_4_^+^ cations on the fluorine sites, which are hydrogen-bonded to adjacent S^2−^ anions. For the inverse molecular ratio, the ADS phase, a monoclinic *P2*_*1*_*/m* phase can be characterized as (NH_4_)^+^(HS)^−^(H_2_S) (Fig. 2g). The (HSH)…(SH) sublattice forms a hydrogen-bonded network. As noted, both AHS and ADS quickly become unstable under pressure.

In contrast, AQS emerges as a very stable mixture, firstly with a *P2*_*1*_*/m* phase (Fig. 2h) that has S^2−^ anions surrounded by (NH_4_)^+^ and NH_3_. It converts to a higher-symmetry tetragonal *I4/m* phase (Fig. 2i) at 58 GPa. The tetragonal phase features S^2−^ anions and the unusual (N_2_H_7_)^+^ cation, which was also reported in (NH_3_)_4_(H_2_O)^21^ and is known from ammonium iodide salts^48^. AQS then decomposes into AMS and S at 83 GPa. However, when pressure is increased up to 300 GPa, a re-stabilized mixture in a triclinic *P-1* phase (Fig. 2j) should be recovered from NH_3_, H_3_S and S. This re-emergent phase remains stable up to 525 GPa. The chemical bonding in this high-pressure phase is completely different from the low-pressure polymorphs of AQS. The (N_2_H_7_)^+^ cation is replaced by ammonium, (NH_4_)^+^; the S^2−^ anion is replaced by (HN-(SH_4_)-NH)^2−^ clusters, with a central S atom that is connected to four H atoms and two (NH) groups.

### Equation-of-state

**Fig. 3:**
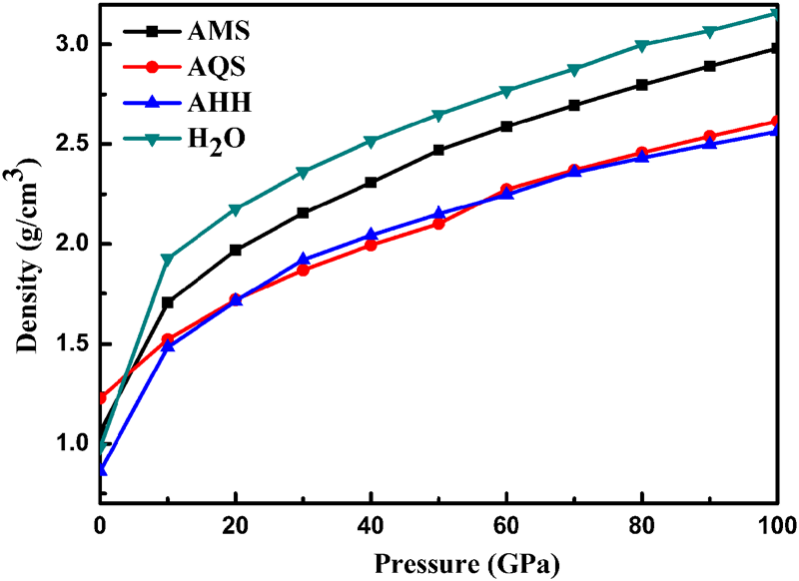
Gravimetric densities of various icy mixtures as function of pressure. Mass densities up to 100 GPa for best crystal structures of 1:1-H_2_S-NH_3_ (AMS), 1:4-H_2_S-NH_3_ (AQS), 1:2-H_2_O-NH_3_ (AHH), and pure ice (H_2_O).

Previous calculations on ammonia hydrates suggested that ammonia-rich hydrate could precipitate out of any ammonia-water mixture and form a layer above a water-rich ocean in icy planets^42^. Our equation-of-state calculations (Fig. 3) suggest that the AMS compound would be situated between those layers; for instance, at 80 GPa, AMS has a gravimetric density of 2.79 g/cm^3^, which is heavier than ammonia hemihydrate (2.44 g/cm^3^) and lighter than ice VIII/X (2.99 g/cm^3^). This suggests hydrogen sulfide could be bound in stable ionic compounds with ammonia in reservoirs towards the top of the icy planets’ mantle regions, but additional work on the thermal equation-of-state is needed to confirm this.

## Discussion

### Chemical bonding and redox reversal

**Fig. 4:**
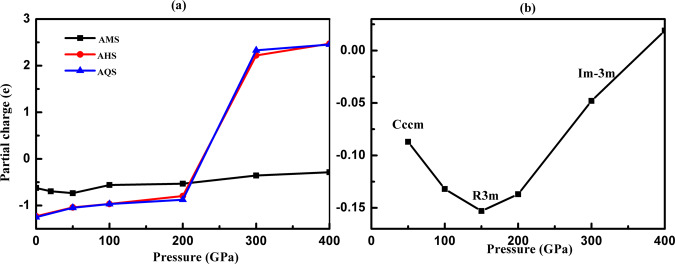
Bader partial charge analyses. **a** Partial charges of sulfur atom s in different H_2_S-NH_3_ mixtures. **b** Partial charges of sulfur atoms in H_3_S, for different solid phases as labeled.

At low pressures, below around 1Mbar, the hydrogen sulfide-ammonia mixtures adhere to the expected chemical picture: H_2_S molecules are likely to disintegrate, with protons transferring to NH_3_ molecules as much as possible. As a consequence, ionic structures form (with isolated S^2−^ anions in ammonia-rich phases) that are further stabilized by hydrogen bonding. These interpretations are supported by topological analyses of the electronic charge density using Bader’s Quantum Theory of Atoms in Molecules (QTAIM) approach, and the electron localization function (ELF). ELF isosurface plots are compiled in Supplementary Figs. 16–18 and Bader partial charges tabulated in Supplementary Tables 6–8. Partial charges of sulfur atoms are also shown in Fig. 4. For sulfur in isolated S^2−^ or SH^−^ anions they range from −0.62 to −1.23 in AMS, AHS and AQS, consistent with a formal oxidation state −2.

However, at high pressures, and led by the re-emergence of stable AQS, an interesting reversal of chemistry occurs. Structurally, sulfur evolves from an isolated anion at low pressure to instead form the central atom of different octahedral clusters at high pressure. In *P-1*-AQS, sulfur forms [SH_4_(NH)_2_]^2−^ clusters that are packed together with NH_4_^+^ cations. In both AHS and AMS at high pressures, the most stable phases (though metastable against decomposition) also feature sulfur at the centers of -(SH_4_N_2_)- and -(SH_2_N_2_)- clusters, respectively. Only in ADS, the most H_2_S-rich compound studied here, did we not find such a role reversal for sulfur (see Supplementary Figs. 19–21). Partial charges from the Bader analysis (see Fig. 4 and Supplementary Tables 6–8) suggest drastic changes in the character of sulfur and hydrogen. For *P2*_*1*_*/m*-AQS at 20 GPa and *I4/m*-AQS at 70 GPa, every hydrogen donates about 0.43e; both N and S act as electron acceptors, with partial charges of −1.21e (−1.22e) and −1.12e (−1.01e), respectively. When pressure increases up to 400 GPa, in *P-1*-AQS, S instead *donates* 2.45e while the terminal H atoms *accept* about 0.26e in the formation of (HN)^2−^-(SH_4_)^2+^-(NH)^2−^. The partial charge on S suggests a drastic change of oxidation state, not inconsistent with S^6+^, while H is in the form of hydride H^−^. Nitrogen is largely unaffected: there are two types of N atoms in *P-1*-AQS that form NH^2−^ and NH_4_^+^ groups, and which accept about 1.64e and 1.27e, respectively.

Sulfur and hydrogen demonstrate an extraordinary capacity to switch from oxidizing to reducing agent, and vice versa. The same reversal in charge transfer can be seen in solid H_3_S itself (see Fig. 4), albeit on a much smaller numerical scale: in the low-pressure molecular *Cccm* and *R3m* phases of H_3_S, sulfur is a weak electron acceptor, but within the atomic (and most relevant superconducting) *Im-3m* phase it eventually (at 400 GPa) transforms into a weak electron donor. It is possible that this redox reversal governs the stability of the sulfur hydride mixtures: the S–H bonding should be weakest around the pressure where the charge transfer is reversed, and the mixtures that are stable in the low pressure regime (where sulfur occurs as S^2−^) should be destabilized in an intermediate pressure regime before re-stabilizing at very high pressures (with sulfur as S^6+^). This is what is seen in all but the most sulfur-rich mixtures.

**Fig. 5:**
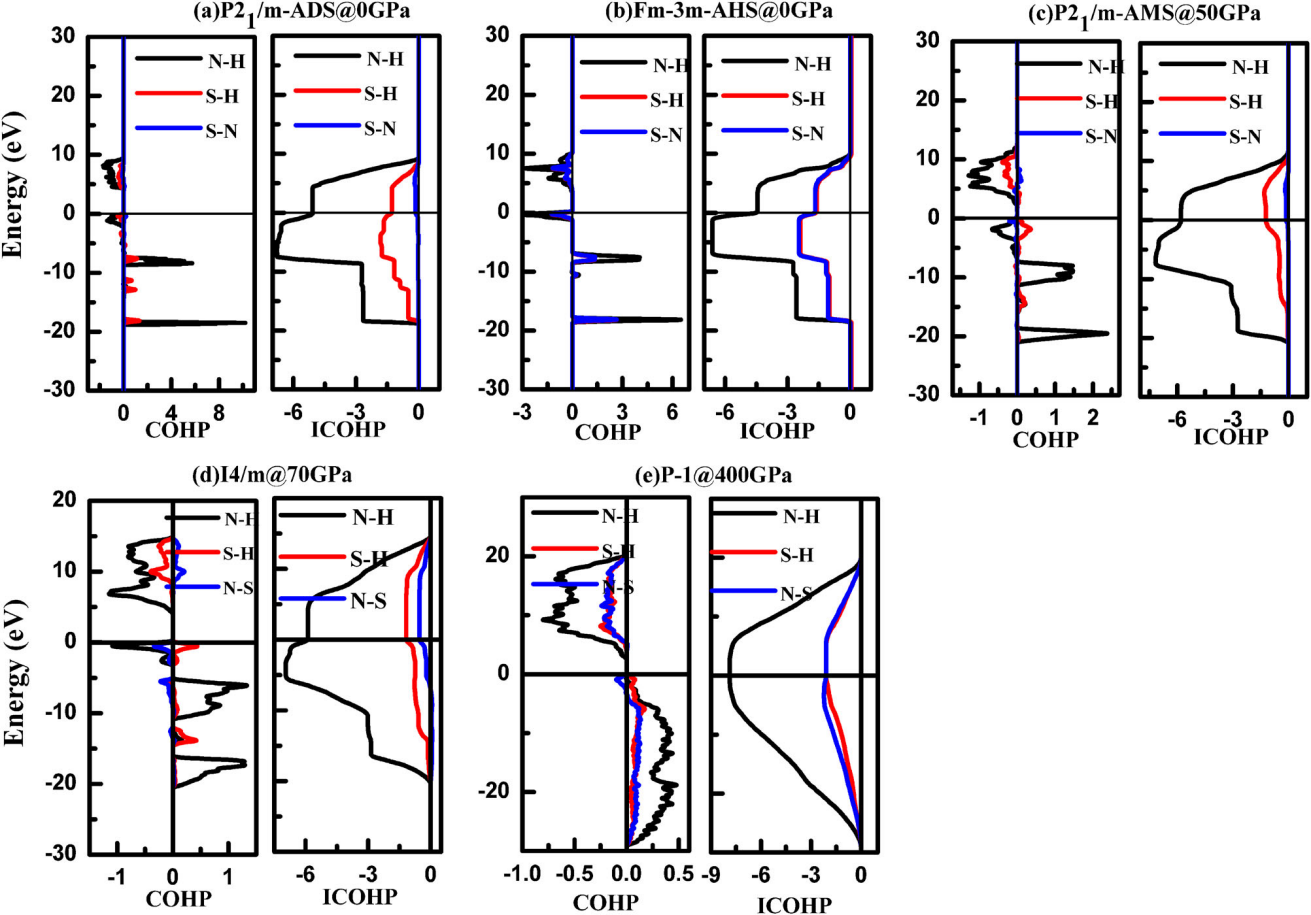
Crystal overlap Hamilton population analyses of different mixtures under pressure. The COHP and integrated COHP (ICOHP) as function of energy shown for **a** *P2*_*1*_*/m*-ADS at 0 GPa, **b** *Fm-3m*-AHS at 0 GPa, **c** *P2*_*1*_*/m*-AMS at 50 GPa, **d** *I4/m*-AQS at 70 GPa, **e** *P-1*-AQS at 400 GPa.

To further characterize the nature of the bonding in the mixtures, we calculated the Crystal Orbital Hamilton Population (COHP) and integrated COHP (ICOHP). By projecting Kohn-Sham Bloch states onto pairs of atom-centered orbitals, the COHP and ICOHP give an indication of band energy-resolved and total chemical bonding strength between pairs of atoms. A *negative* COHP below the Fermi level indicates bonding states, whereas *positive* COHP indicates anti-bonding states. Figure 5 shows the average COHP and ICOHP of nearest-neighbor N–H, S–H and S–N interactions for representative mixture phases. In all structures covalent N–H bonding is most prominent (despite partially occupied anti-bonding states in NH_4_^+^ and N_2_H_7_^+^ cations). In contrast, in the low-pressure phases, both S–H and S–N bonding are relatively weak—consistent with having isolated S^2−^ anions hydrogen-bonded to NH_4_ or N_2_H_7_. In the high-pressure *P-1*-AQS phase, S–H (S–N) bonds between the nearest neighboring S–H (S–N) pairs also have smaller COHP values than N–H (Fig. 5e), indicating weaker covalent bonding, but are much larger than in the low-pressure *I4/m* phase: the average ICOHP for each S–H and S–N bond is −2.1 eV in the *P-1* phase, compared to −1.17 and −0.53 eV in *I4/m*. Furthermore, the nearest S–H (1.84 Å) and S–N (2.82 Å) distances in the *I4/m* phase at 70 GPa are both much larger than in the *P-1* phase (1.28 and 1.52 Å, respectively); this compares to covalent S–H and S–N bond lengths of 1.59 Å in H_3_S at 40GPa^1^ and 1.70 Å in solid N_4_S_4_ at ambient pressure^49^. By comparison, the S–H bonding in pure H_3_S is relatively weak (see Supplementary Fig. 22, ICOHP = −0.5 eV at 400 GPa), which illustrates how the ad-mixture of NH_3_ can improve the strengths of H-S or N-S bonds greatly.

**Fig. 6:**
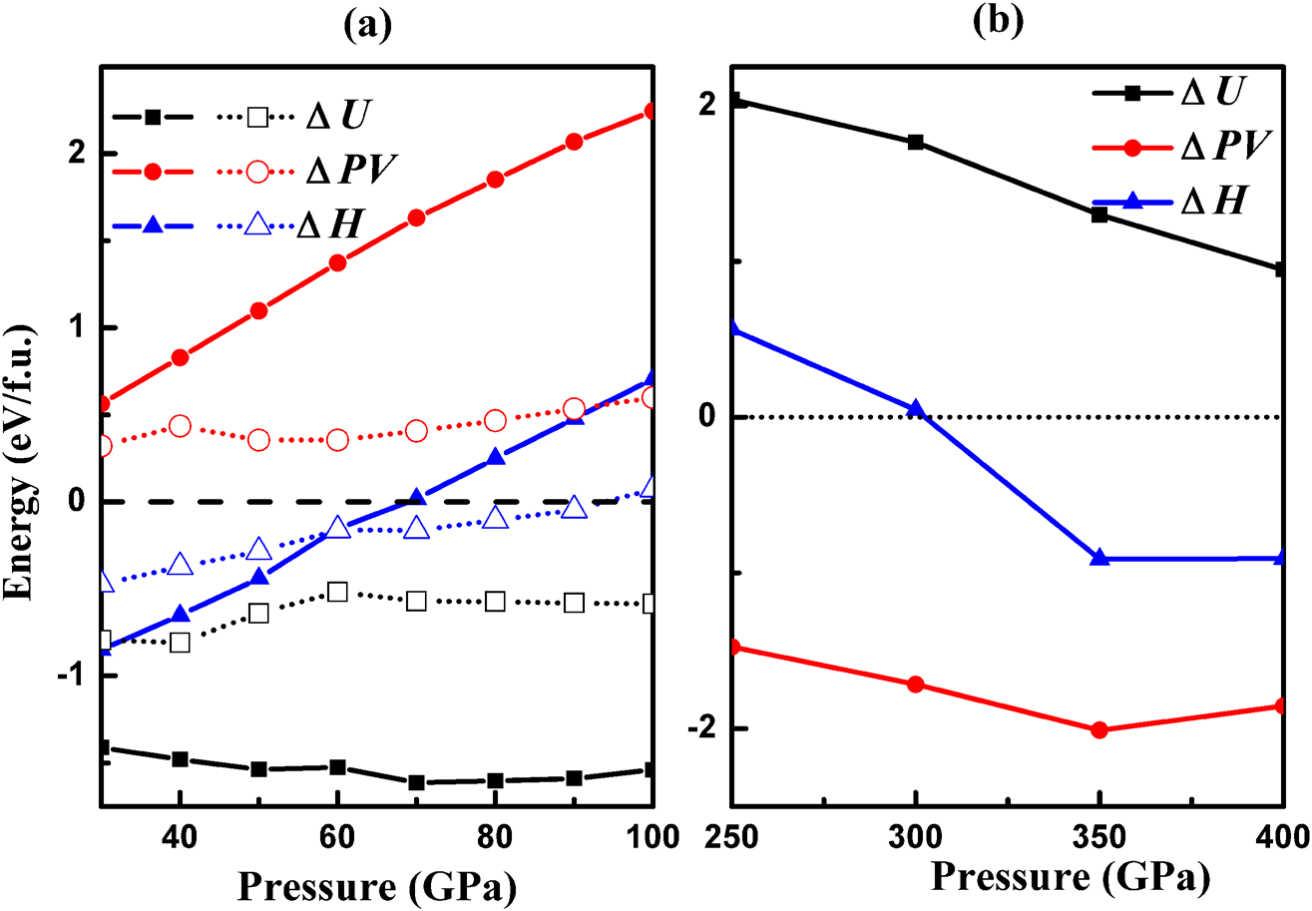
Calculated Δ*H*, Δ*U*, and Δ(*PV*) for 1:4-H_2_S-NH_3_ phases as a function of pressure. **a** Low pressure phases *P2*_*1*_*/m* (solid lines) and *I4/m* (dotted lines). **b** High pressure *P-1* phase. Both graphs are relative to the decomposition reaction 4NH_3_ + (2H_3_S + S)/3.

Finally, to further illuminate why pressure is able to induce those changes, we examined the evolution of the contributions to the formation enthalpy *H* = *U* + *PV*, the internal energy (*U*) and the product of pressure and volume (*PV*), for the stable AQS phases in response to pressure change. In Fig. 6a, the pressure dependence of Δ*U*, Δ(*PV*), and Δ*H* for the low-pressure *P2*_*1*_*/m* and *I4/m* phases are shown relative to the reference decomposition 4*NH_3_ + (2H_3_S + S)/3. Both compounds are stabilized by internal energy against decomposition (likely due to ionic bonding), whereas the Δ(*PV*) term is positive for both. *I4/m*-AQS is more compact, which leads to the phase transition from *P2*_*1*_*/m* to *I4/m* at 58 GPa. Above 58 GPa, the Δ*U* and Δ(*PV*) terms for *I4/m* are relatively well balanced, resulting ultimately in decomposition of AQS at 83 GPa into NH_3_, H_3_S and S. For the high-pressure *P-1*-AQS phase (Fig. 6b) the picture has reversed: the Δ(*PV*) term is strongly negative while Δ*U* is positive. The re-emergence of AQS at 300 GPa is due to its more compact structure that centers around the small S cation, which outweighs the electronic cost of ionizing the sulfur 3p shell.

### Electronic properties

**Fig. 7:**
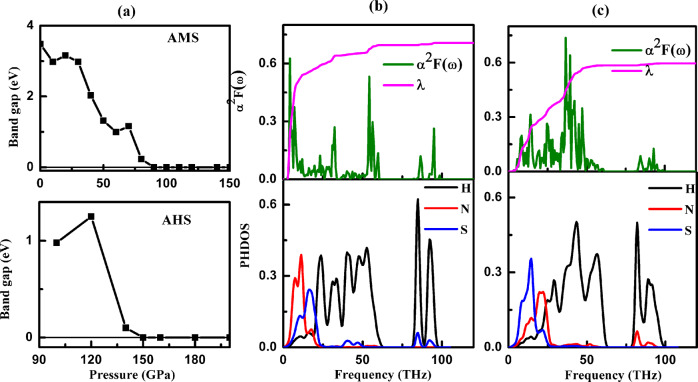
Electronic properties of different mixtures. **a** Band gap evolution with pressure across best structures of AMS and AHS mixtures. **b**–**c** Eliashberg spectral function α^2^F(ω), integrated electron–phonon coupling parameter λ and projected phonon densities of state (PHDOS) for **b** *Abm2*-AMS at 100 GPa, **c** *Cmma*-AMS at 200 GPa.

Molecular “hot ice” mixtures are usually electronically not very interesting wide gap insulators and require very extreme conditions of pressure and/or temperature to close the band gaps. However, H_2_S and H_3_S metallize at relatively low pressures, below 1 Mbar. This property seems to translate to their mixtures with methane, CH_4_, ultimately resulting in a room temperature superconducting material at 250 GPa^9^. Therefore, the electronic structures of all the stable mixtures found here were calculated within their stability ranges, and pressure evolutions of their electronic band gaps are shown in Fig. 7a (partial densities of states (DOS) are compiled in Supplementary Figs. 23–26). The band gap data shows that ADS, AHS and AQS all retain wide band gaps below 100 GPa. For AMS, the *P*4*/nmm*, *Cc* and *P*2_1_*/m* phases all have wide band gaps below 90 GPa. At higher pressure we observe that *Abm*2-AMS and *Cmma*-AMS exhibit metallic character. In our calculations *Abm2*-AMS experiences band gap closure from semiconductor to metal at about 88 GPa, possibly underestimated at DFT-PBE level of theory, and at the upper end of its range of stability. The *Cmma*-AMS phase is metallic in its full range of stability. To find metallic molecular mixtures at these relatively low pressures remains unusual. A metallic fluid of H_2_–H_2_O was predicted above 5000 K and 500 GPa^26^ while hot dense hydrogen seems to require at least 1500 K at 150 GPa^50,51^. On the other hand, H_2_S itself metallizes at 96 GPa^52,53^, an unknown carbonaceous H_2_S material was reported to metallize at 60 GPa^9^, and metastable CH_4_–H_2_S mixtures were predicted to be metallic at 100 GPa and above^7,8^. Here, we find that specific stable mixtures of H_2_S with ammonia retain this property as they approach the intermediate pressure regime—where low binding energies and delocalized electronic states can both be explained by the redox reversal induced in sulfur and hydrogen.

The Fermi energies for the *Abm*2 phase at 100 GPa and *Cmma* phase at 200 GPa (Supplementary Fig. 24d, e) are located at shoulders of valence peaks in the DOS that are dominated by N-2p and S-3p states, leading to non-negligible values of the DOS at the Fermi energy in particular for *Cmma*-AMS. These features are potentially favorable for high-temperature superconductivity. We therefore calculated the electron–phonon coupling to investigate the superconductivity of these high-pressure phases of AMS. Figure 7b, c shows the projected phonon density of states (PHDOS), Eliashberg spectral function α^2^F(ω), and electron–phonon coupling integral λ(ω) for *Abm2*- and *Cmma*-AMS under pressure. A notable feature of phonon density of states for both phases is the separation of the vibrational modes into three distinct regions: The lower frequencies (below 20 THz) are associated with lattice modes of the heavier S and N atoms, and some of hydrogen atoms; the intermediate frequencies (between 20 and 60 THz) are mainly derived from combinations of molecular libration, bending, and stretching modes; and the highest frequencies (80∼100 THz) are derived primarily from the N–H stretching modes of the molecular NH_4_ units. The contribution to λ(ω) is mainly from the low frequency (0–20 THz, about 56.8%) and mid-frequency region (20–60 THz, about 34.1%), while the highest vibrons (80∼100 THz) contribute about 9.1%. The superconducting temperatures are estimated by the Allen-Dynes modified McMillan equation^54^, using a typical value (μ^*^=0.1) of the Coulomb pseudopotential. The calculated values of *λ*, *ω*_log_ and *T*_c_ of the *Abm2* and *Cmma* phases of AMS under pressure are shown in Table 1. The *Cmma*-AMS phase at 150 GPa has the largest *λ* value of 0.995 and with *ω*_log_ = 697 K, leads to a *T*_c_ of 49.9 K. This represents the largest *T*_*c*_ found here, as further compression results in a decrease of *T*_*c*_. Supplementary Table 10 lists predicted *T*_*c*_ values for other, metastable, phases, which can also reach up to 46 K (in ADS at 300 GPa).

**Table 1:** Calculated EPC parameter (λ), logarithmic average phonon frequency (ω_log_) and superconducting temperature (T_c_) for stable phases of AMS at the pressures given.

Phase	Pressure (GPa)	λ	ω_log_ (K)	*T*_c_ (K)
*Abm2*	100	0.703	398	13.9
	120	0.772	493	21.4
*Cmma*	150	0.995	697	49.9
	200	0.625	987	27.9

## Conclusions

In summary, we performed an extensive structure search in combination with first-principles total energy calculations to explore the formation, stability, chemical bonding and electronic properties of nitric sulfur hydrides at high pressure, as formed from mixtures of H_2_S and NH_3_. While only two mixed compounds, NH_4_SH and (NH_4_)_2_S, are known to exist at or near ambient conditions (and we confirm their stability) we report a series of new phases across four different stoichiometries with very different high-pressure properties. Two compounds, ADS and AHS (the 1:2 and 2:1 mixtures), are stable only at very low pressures, while AMS (1:1) is stable at moderate pressure in a series of different phases up to 129 GPa. AQS (ammonia-rich 4:1) is initially stable at pressures below 83 GPa. However, it becomes stable again under higher pressure above 300 GPa and remains such up to 525 GPa through an interesting reversal of the sulfur chemistry.

At low pressures, hydrogen sulfide disintegrates very rapidly, with the sequence H_2_S → (HS)^−^ → S^2−^ that can be triggered as low as ambient pressure; accordingly, the compounds feature NH_3_, NH_4_^+^, and N_2_H_7_^+^ species, depending on stoichiometry. The ionic bonding helps to stabilize these structures and all elements are, corroborated by QTAIM analysis, in expected oxidation states (S^2−^, N^3−^, and H^+^) that correlate with their electronegativities (Pauling scale: 2.58/3.04/2.20 for S/N/H). At high pressures, it is instead advantageous to *oxidize* sulfur, as it becomes the central atom in clusters of the form: N-(SH_4_)-N or HN-(SH_4_)-NH. This is balanced by the presence of hydride H^−^ while N^3−^ remains as an anion. This partial reversal in redox potential is in line with a recent computational estimation of atomic electronegativities at high pressure (at 300 GPa: 5.8/10.1/6.1 for S/N/H)^55^ but still represents a striking reversal of chemistry. It occurs in most mixtures (except the most sulfur-rich) but only for AQS results in a stable phase; a breakdown of enthalpy contributions shows that the resulting structure is much more compact, likely due to octahedron formation around the S^6+^ cation, which outweighs the electronic cost of ionizing the S-3p shell. In compressed H_3_S, where one might consider sulfur to be octahedrally coordinated, a similar reversal of charge transfer occurs, albeit on a much smaller scale, possibly due to effective screening by the conduction electron sea.

Most stable compounds discussed here are wide-gap insulators or semiconductors. Interestingly, however, around 100 GPa pressure a few compounds exhibit insulator-metal transitions. Electron–phonon coupling calculations indicate that these compounds can support electron–phonon mediated superconductivity up to about 50 K. Metallization occurs due to phase transitions into inherently metallic phases and is therefore not likely to be affected by DFT’s underestimation of the electronic band gap. These metallic phases are favored in an intermediate pressure regime where the redox reversal is imminent, leading to much less ionic character and diminished binding energies. In carbonaceous H_2_S, metallization was reported already at 60 GPa^9^. It remains unusual to find dense molecular mixtures that are metallic at less than very extreme temperature conditions: even above the melting line they tend to remain insulating until heated to much higher temperatures^15^. Conductivity in ‘hot ices’ along typical planetary isentropes is therefore predominantly of ionic origin. However, when conducting states are eventually reached, the electronic conductivity can dominate ionic contributions by several orders of magnitudes^56^. Here, we present examples of stable molecular mixtures that are metallic in the ground state and will remain so at all elevated temperature conditions.

Finally, the existence of stable hydrogen sulfide-ammonia phases to 1 Mbar and beyond suggests that H_2_S can be bound deep inside icy planets. Equation-of-state calculations suggest it might accumulate in a thin layer between ammonia hydrates and an ice-rich ocean. However, H_2_S remains a minority component in icy planet mantles and the enthalpic stabilization of mixtures such as discussed here must be tempered by entropic stabilization of dissolving H_2_S in water-rich solutions. Nonetheless, its potential presence in deep planetary reservoirs should be considered when interpreting atmospheric measurements. The bifurcation of the stability regions of nitric sulfur hydrides, driven by two distinct sulfur-hydrogen chemistries, suggests that these mixtures could also be present towards the bottom of the icy ocean, resulting in an unusual stratification of compounds that share the same constituents.

## Methods

### Structural predictions

Structure searches for energetically stable crystalline structures were performed on various stoichiometries of (H_2_S)_*x*_(NH_3_)_*y*_ from *x*:*y* = 1:4 to 2:1 using simulation cells containing up to four formula units. Structure searches for all stoichiometries were carried out at 10 GPa, 50 GPa, and from 100 GPa to 800 GPa in increments of 100 GPa by particle swarm optimization methodology as implemented in the CALYPSO code^57,58^. In addition, datasets were augmented by structures drawn from the analogous (H_2_O)_*x*_(NH_3_)_*y*_ system^21^. The CALYPSO methodology is highly effective in finding stable or metastable structures only depending on the given chemical composition and external conditions, and has been applied successfully to various elemental solids, binary, and ternary compounds^59–62^.

### Ab initio calculations

Structural optimizations and electronic structure calculations were performed in the DFT framework with the Perdew−Burke−Ernzerhof (PBE)^63^ exchange-correlation functional, as implemented in the Vienna Ab initio Simulation Package code^64^. Their reliability was confirmed with all-electron calculations, see Supplementary Discussion and Supplementary Fig. 1. The calculation of phonon-mediated superconductivity was performed with the Quantum-ESPRESSO package^65^. More detailed computational information can be found in the Supplementary Methods, section ‘Computational Details’.

### Phase stability calculations

Specifically, and motivated by preceding studies of the NH_3_-H_2_O system^42^, the mixtures studied here are ammonia mono-sulfide (AMS, NH_3_:H_2_S = 1:1), ammonia di-sulfide (ADS, 1:2), ammonia hemi-sulfide (AHS, 2:1), ammonia tri-sulfide (ATS, 3:1), and ammonia quarter-sulfide (AQS, 4:1). The known compounds NH_4_SH and (NH_4_)_2_S fall under the AMS and AHS stoichiometries, respectively. The formation enthalpy of each H_2_S-NH_3_ compound is defined as $H = H{[ {( {{\mathrm{H}}_2{\mathrm{S}}} )_x( {{\mathrm{NH}}_3} )_{y}]} - xH( {{\mathrm{H}}_2{\mathrm{S}}} ) - yH({\mathrm{NH}}_3)}$, where the most stable *P2*_*1*_*3* and *P2*_*1*_*2*_*1*_*2*_*1*_ phase of NH_3_ and the *Pbcm* and *P2/c* phases of H_2_S at the lower pressure below 60 GPa were considered. When pressure increases beyond 60 GPa, H_2_S was identified to decompose into H_3_S and S, and the formation enthalpies of H_2_S-NH_3_ above 60 GPa were calculated relative to the most stable phases of H_3_S and S^2^ and NH_3_ (*Pma2* and *Pca2*_*1*_)^66^.

### Chemical bonding analyses

Chemical bonding analyses utilized Bader’s QTAIM approach^67^ as implemented in the CRITIC2 code (see Supplementary Tables 6–9 in the Supplementary Discussion)^68^, the electron localization function (ELF)^69^, and the Crystal Orbital Hamilton Population (COHP) analysis^70^ as implemented in the LOBSTER code^71,72^. The phonon dispersion curves of stable compounds as shown in Supplementary Figs. 2–5 in the Supplementary Discussion, are calculated at their stable pressure range^73,74^, from which it becomes clear that all of the mixtures in the H_2_S-NH_3_ system discussed below are dynamically stable due to the absence of imaginary phonon frequencies. Detailed information on the predicted stoichiometries/structures is presented in the Supplementary Discussion, section ‘Crystallographic Information’, Supplementary Tables 1–5.

## Supplementary information

Supplementary Information

## Data Availability

The data that support the findings of this study are available from the authors upon reasonable request.
