# Supplementary material for: First principles study of dense and metallic nitric sulfur hydrides
Source: Commun Chem. 2021 Jun 4;4:83. doi: 10.1038/s42004-021-00517-y (PMC9814481; doi:10.1038/s42004-021-00517-y)
Supplement: Supplementary file 1 — Supplementary Information [file 42004_2021_517_MOESM1_ESM.pdf]

SUPPLEMENTARY INFORMATION

First principles study of dense and metallic nitric sulfur hydrides

Xiaofeng Li[†, ‡], Angus Lowe[‡], Lewis Conway[‡], Maosheng Miao[§, #], Andreas Hermann[‡, *]

[†]College of Physics and Electronic Information, Luoyang Normal University, Luoyang 471934, P. R. China

[‡]Centre for Science at Extreme Conditions and SUPA, School of Physics and Astronomy, The University of Edinburgh, Edinburgh EH9 3FD, United Kingdom

[§]Department of Chemistry & Biochemistry, California State University, Northridge, CA, 91330-8262, USA.

[#] Department of Earth Science, University of California Santa Barbara, CA, USA

[*] Email: a.hermann@ed.ac.uk

SUPPLEMENTARY METHODS **2**

COMPUTATIONAL DETAILS 2

SUPPLEMENTARY DISCUSSION **2**

ALL ELECTRON VS PSEUDOPOTENTIAL CALCULATIONS 2

HIGH-PRESSURE PHASES OF METASTABLE NH_3-H_2S COMPOUNDS 3

SULFUR POLYHEDRAL PHASES 5

PHONON DISPERSION CURVES 7

RELATIVE FORMATION ENTHALPIES 8

ZERO-POINT ENERGY CORRECTED CONVEX HULLS 9

FORMATION OF NH_3-H_3S-S COMPOUNDS 10

PHASE STABILITY AND PROPERTIES OF ATS UNDER PRESSURE 10

ELECTRON LOCALIZATION FUNCTION (ELF) 12

CRYSTAL STRUCTURE REPRESENTATIONS 13

CHARGE TRANSFER AND COHP IN H_3S 14

PARTIAL DENSITIES OF STATE (PDOS) 14

CRYSTALLOGRAPHIC INFORMATION 17

BADER CHARGE TRANSFER 23

SUPERCONDUCTING TEMPERATURES AND RELEVANT PARAMETERS 25

SUPPLEMENTARY REFERENCES **26**

Supplementary Methods

Computational Details

Structural optimizations and electronic structure calculations were performed in the DFT framework with the Perdew−Burke−Ernzerhof (PBE)[1] parametrization of generalized gradient approximation, as implemented in the Vienna Ab initio Simulation Package code.[2] The $1s^1$ (H), $2s^22p^3$ (N), and $3s^23p^4$ (S) electrons were treated explicitly as valence electrons. High quality computational parameters were used when evaluating the enthalpies of structures during the searches. A plane-wave basis set cutoff of 1200 eV and Monkhorst−Pack scheme with a dense k-point grid of spacing $2\pi \times 0.03$ Å^{-1} in Brillouin zone were found to give converged enthalpies within 1 meV/atom. To determine dynamical stabilities, phonon calculations were performed by using a supercell approach with the finite displacement method[3] and the Phonopy code,[4] which uses the Hellmann−Feynman forces calculated from the optimized supercell using DFT as implemented in the VASP code.[2] Electron localization function (ELF) and Bader QTAIM analyses were used to investigate the chemical bonding.[5]

Electron−phonon coupling (EPC) and superconducting properties were calculated using density function perturbation theory as implemented within the framework of linear response theory through the Quantum-ESPRESSO code.[6] Ultrasoft pseudopotentials for the N, S, and H elements were used with a kinetic energy cutoff of 90 Ry. A q-mesh of 5×5×5, 5×5×6, 5×5×4, 6×6×4 and 4×4×4, a k-mesh of 20×20×20, 20×20×24, 20×20×16, 24×24×16 and 16×16×16 for Abm2, Cmma,C2/m, $P2_1/m$- II and $P2_1$ phases of AMS and a q-mesh of 6×3×3, 5×5×3 and 6×3×3, a k-mesh of 24×12×12, 20×20×12 and 24×12×12 for P-1-ADS, Ccc2-AHS and P-1-ATS respectively, were used in the electron-phonon coupling calculations. The superconducting temperature T_c of ADS, AMS, AHS and ATS were calculated via the Allen −Dynes-modified McMillan equation.[7]

Supplementary Discussion

All Electron vs Pseudopotential Calculations

In order to examine the reliability of the adopted projected-augmented-wave (PAW) pseudopotentials at high pressures for S, N and H, we compare it to the all-election linear augmented plane wave (LAPW) method as implemented in the WIEN2k code.[8] By using the two different methods, total energies of $(NH_3)_4(H_2S)$ in the P-1 structure are calculated at high pressures, and then

fit the obtained energy-volume data into the Birch-Murnaghan equation of states. Supplementary Fig. 1 shows the resulted fitted equation of states. The results obtained from two methods are almost identical, which clearly indicates the suitability of the PAW pseudopotentials for describing the energetics of NH_3-H_2S compounds at pressures.

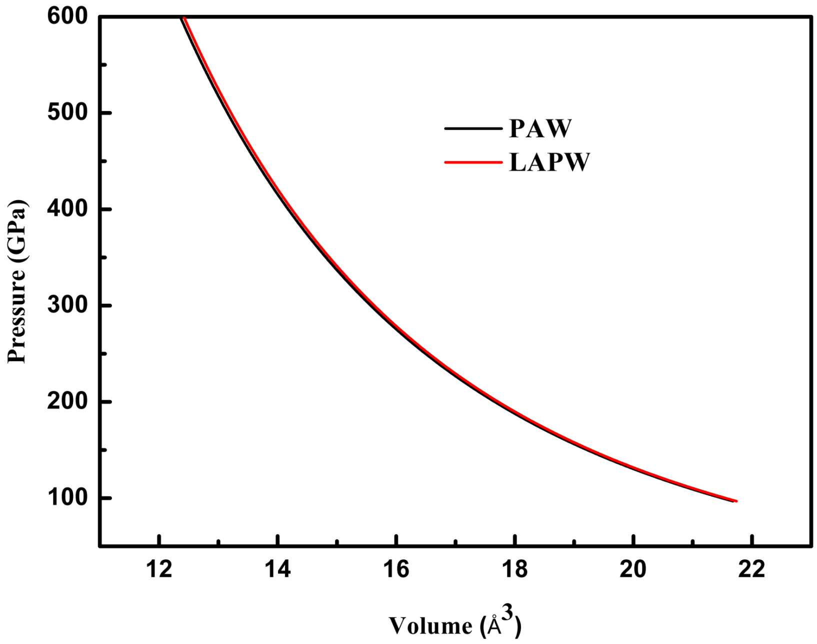

Supplementary Figure 1. Comparison of the fitted Birch-Murnaghan equation of states for $(NH_3)_4(H_2S)$ in the P-1 phase by using the calculated results from the PAW pseudopotentials and the full-potential LAPW methods

High-pressure phases of metastable NH_3-H_2S compounds

For the NH_3-H_2S system under pressure, complicated phases exist under higher pressure.

Ammonia di-sulfide

ADS is a rich-H_2S compound and has an important significance for understanding icy planets at extreme conditions. At ambient condition, a monoclinic $P2_1/m$ phase become a stable phase at ambient condition, which transformed to another phase $P2_12_12_1$ (see Supplementary Fig. 6) at 60GPa. Under higher pressure above 207GPa, a new monoclinic phase P-1 phase is energetically favored. Three phases had similar structures and exhibited half-ionicity as $(NH_4)^+(HS)^-(H_2S)$, which is the same to that of ADH (8). In three structures, every N atom donates four hydrogen bonds and H_2S loses a hydrogen atom and converted into HS^- unit (see Supplementary Fig. 26). ELF of three phases (see Supplementary Fig. 16) also established their chemical bonding characterizations.

Moreover, the electronic structure calculation showed that $P2_1/m$ and $P2_12_12_1$ phases have wide band gap under pressure. Interestingly, P-1 phase exhibited obvious metallicity under pressure (see Supplementary Fig. 22). Excitingly the obtained superconducting temperature of P-1 phase at 300GPa is about 46.4K which is close to that of Cmma-AMS at 150GPa (see Supplementary Table 10).

Ammonia mono-sulfide

As in AMH, from 0GPa to 200GPa there are rich phases which evolve from simple ionic $(SH)^-$ to one dimensional infinite chain of linear S-H-S bond, and to chain of zigzag S-H-S bond (see Supplementary Fig. 20) . Moreover, under higher pressure below 674GPa (see Supplementary Fig. 9), two new monoclinic $P2_1/m$- Ⅰ and C2/m are disclosed which also have a chain formed by zig-zag S-H-S bond same to that under low pressure. When pressure continues to increase, the new monoclinic $P2_1$ phase becomes energetically favored. In this crystal structure (see Supplementary Fig. 20), there are two different chains formed by -(NH)- and -(NH_2)-, respectively, which is connected by the bridge S(H-S-H). The bonding characterization of –(NH_2)- chain also existed in $LiNH_2$. Moreover, there are no imaginary values on phonon curves of C2/m, $P2_1/m$- Ⅰ and $P2_1$ under their corresponding pressure range, which indicated their thermodynamically stable. The calculated density of states of C2/m at 350GPa, $P2_1/m$- Ⅰ at 500GPa and $P2_1$ at 700GPa (see Supplementary Fig. 23) firstly exhibited amazing metallicity. Due to the characterization of density of states, it will favor their superconductivity. The superconducting temperatures T_c, EPC parameter λ and ω_{log} of C2/m at 350GPa, $P2_1/m$-Ⅱ at 500GPa and 600GPa are all calculated, which are all listed in Supplementary Table 10.

Ammonia hemi-sulfides

The energetically favored structure of AHS (see Supplementary Fig. 7) has space group Fm-3m and could form fully ionic $(NH_4)^+S^{2-}$ structures. When pressure is beyond 4GPa, enthalpy of cubic phase increases sharply, and a less symmetric orthorhombic phase Cmcm was favored (see Supplementary Fig. 7). It has more efficient packing, but remains the same features with S^{2-} anions surrounded by NH_4^+ cations that have lots of hydrogen bonds towards the S^{2-}. When pressure increases up to 84GPa, another orthorhombic phase Ccc2 occurs with similar structural features with that of Cmcm phase. Above 201GPa, a new monoclinic phase $P2_1/c$ is stable, and when the pressure is above 726GPa, the monoclinic phase $P2_1/c$ transformed into orthorhombic phase $Pna2_1$. Comparing to the structural

patterns in $P2_1/c$ phase, we can find that -(SH_4N_2)- loses two H atoms and connected two N atoms to form -(SN_4H_2)-, isolated H_2 molecule occurs. Their structural features are exhibited in Supplementary Fig. 19. In order to illustrate their bond characterization, we calculated the electron localization function (ELF) (see Supplementary Fig. 18) which can establish the bonding pattern. The calculated electronic density of states (see Supplementary Fig. 24) show that under low pressure AHS is a semiconductor with a large band gap 3.8eV at 0GPa (Fm-3m phase), 3.1eV at 50GPa (Cmcm phase) and 0.1eV at 150GPa (Ccc2 phase) which indicated the band gap decreases with pressure. In order to study further the electronic structure of Ccc2 under pressure, its band gap decreases down to 0eV at 153GPa and Ccc2 phase has metallicity. Under higher pressure, the density of states of $P2_1/c$ at 300GPa and $Pna2_1$ at 750GPa also indicated that they have bandgap of 2.98eV and 0.98 eV, respectively. So the superconducting temperature of Ccc2 phase at 180GPa is about 4.0K (see Supplementary Table 10).

Ammonia Tri-sulfides

In comparison to NH_3 and H_2S, ATS is metastable against the decomposition of NH_3 and H_2S at low pressure. At ambient condition (see Supplementary Fig. 12), ATS crystallized in a low-symmetry monoclinic half-ionic phase $P2_1$ with NH_3, NH_4^+ and SH^- units (see Supplementary Fig. 13). Above 39GPa, $P2_1$ (see Supplementary Fig. 12) transformed into Pm phase, in which SH^- in $P2_1$ phase converted into S^{2-} anion. When pressure continues up to 365GPa, P-1 phase become energetically favored, which contains NH_3, NH_4^+, linear H_2S and S^{2-} units (see Supplementary Fig. 12). Surely, the bonding patterns of N and H, S and H atoms are verified by ELF (see Supplementary Fig. 13). Though ATS decomposed into NH3, H3S and S under pressure, they are thermodynamically stable (see Supplementary Fig. 14). By the electronic structure calculation (see Supplementary Fig. 15) it indicated their semiconducting characterization for $P2_1/m$ and $P2_12_12_1$ phases. Detailed information on the predicted structural and charge transfer are presented in Supplementary Tables 5-6.

Sulfur polyhedral phases

In AHS, above 201GPa, a new monoclinic phase $P2_1/c$ is stable (though metastable against decomposition), where every sulfur atom has six covalent bonds to form -(SH_4N_2)- units while the nitrogen forms -(NH_2)- zigzag infinite chains. The structural features of AHS-$P2_1/c$ are exhibited in Supplementary Fig. 19 and the ELF (see Supplementary Fig. 18) confirms the interpretation above.

In AMS at 674 GPa a new monoclinic $P2_1$ phase becomes energetically favored (though also metastable against decomposition). In this crystal structure (see Supplementary Fig. 20), there are two different chains formed by -(NH)- and -(NH_2)-, respectively, which are both connected to sulfur. Conversely, here, each S atom is surrounded by two hydrogen and two nitrogen atoms and forms -(SH_2N_2)-. The calculated ELF identifies the bonding for all AMS phases under pressure (see Supplementary Fig. 17).

Full information on the bond critical points (BCP's) in the I4/m and P-1 phases of AQS is given in Supplementary Table 9. This confirms the isolated nature of sulfur in the I4/m phase (low charge density ρ_{BCP}(S…H) = 0.06…0.07 e/Å^3, weakly positive Laplacian $\nabla^2\rho_{BCP}$(S…H) = +0.06…0.09) in contrast to substantial bonding in the P-1 polyhedra (ρ_{BCP}(S-H) = 0.29 e/Å^3, $\nabla^2\rho_{BCP}$(S-H) = -0.98…1.04). The same charge transfer pattern is seen in AHS, where the $P2_1/c$ structure has S and H with partial charges +2.2e and -0.24e (in a SH_4N_2 cluster), and also in AMS, where in the $P2_1$ structure S and H (in a SH_2N_2 cluster) have +1.42e and -0.22e.

Phonon dispersion curves

The phonon dispersion relations for all phases discussed in the main manuscript at pressure points relevant to their respective stability range.

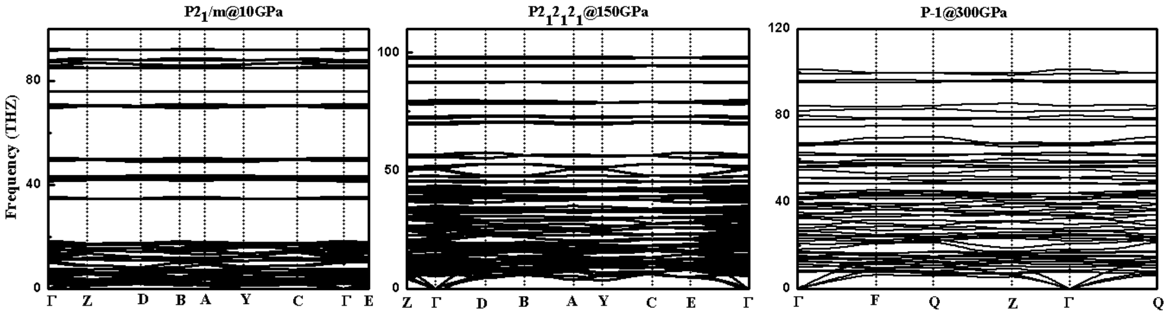

Supplementary Figure 2. Phonon dispersion curves of ADS.

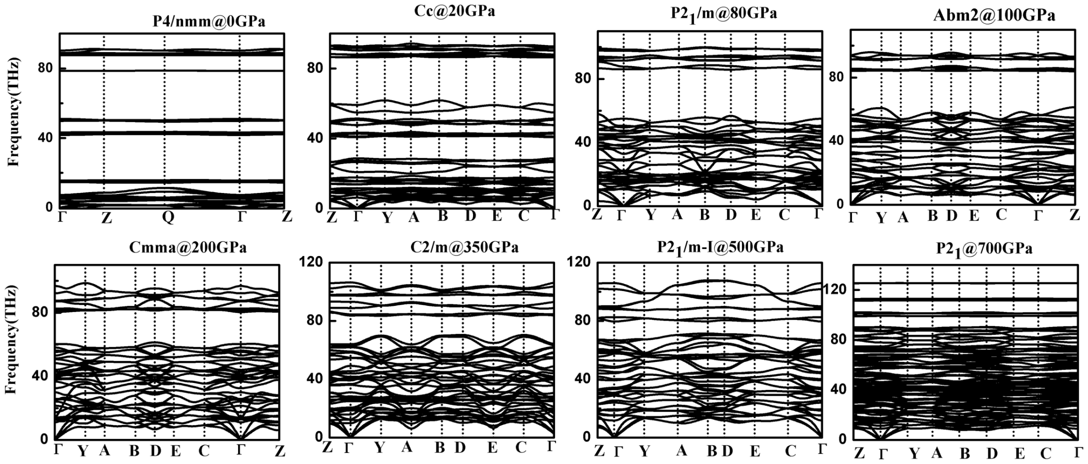

Supplementary Figure 3. Phonon dispersion curves of AMS.

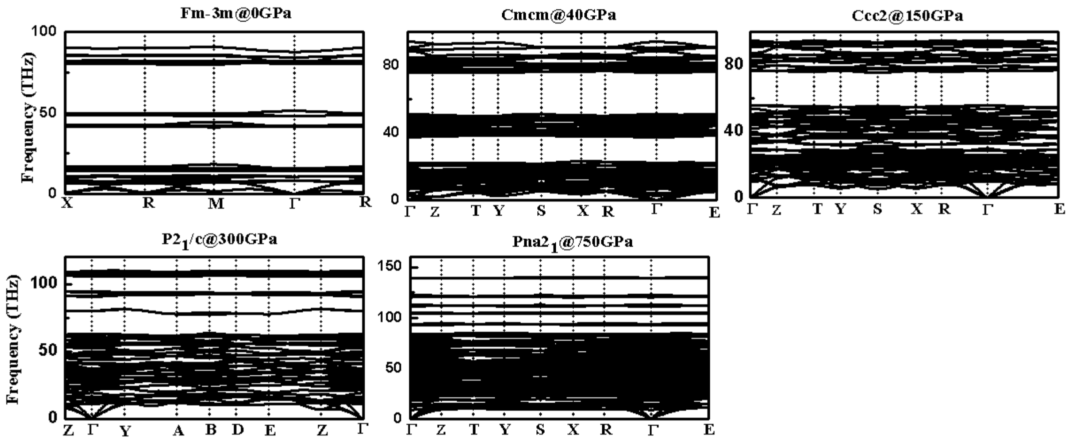

Supplementary Figure 4. Phonon dispersion curves of AHS.

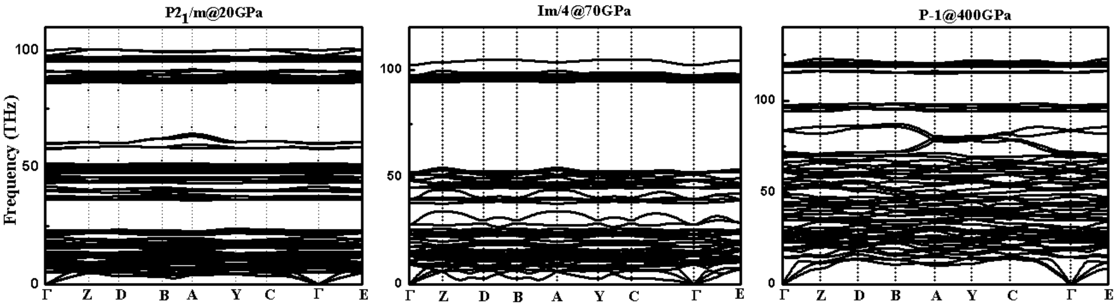

Supplementary Figure 5. Phonon dispersion curves of AQS.

Relative formation enthalpies

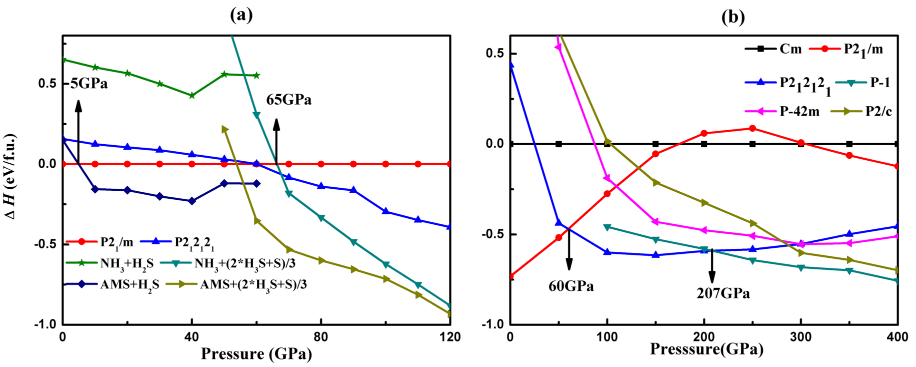

Supplementary Figure 6. The enthalpies variations with pressure for ADS.

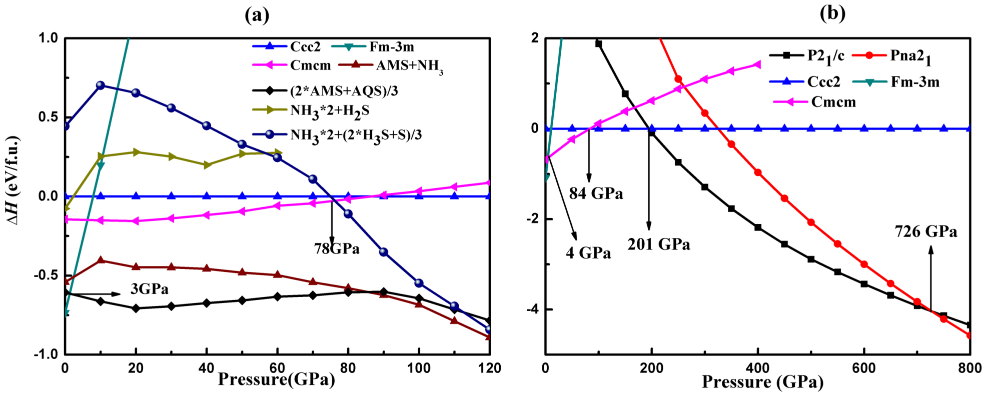

Supplementary Figure 7. The enthalpies relative to *Ccc2* phase of AHS at pressure.

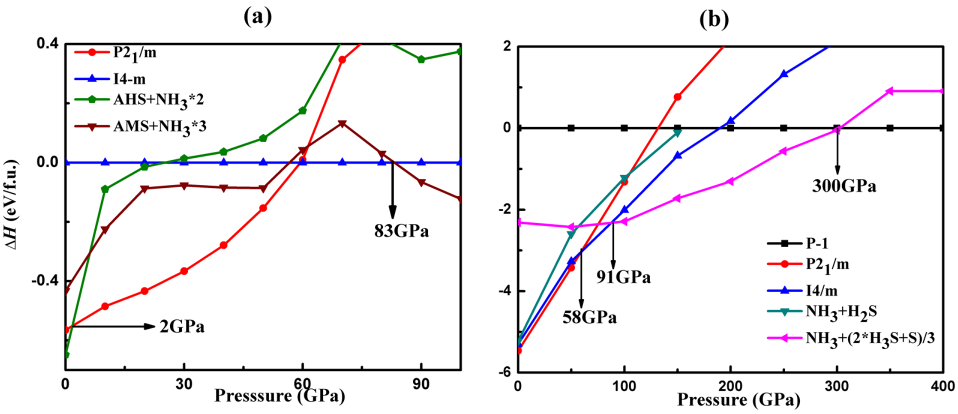

Supplementary Figure 8. The enthalpy differences per formula of AQS as a function of pressure.

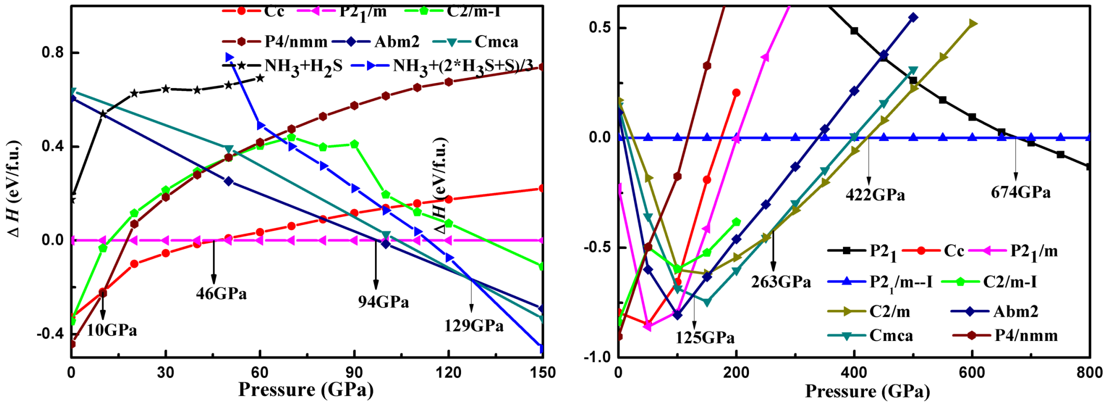

Supplementary Figure 9. The enthalpy differences per formula of AMS as a function of pressure.

Zero-point energy corrected convex hulls

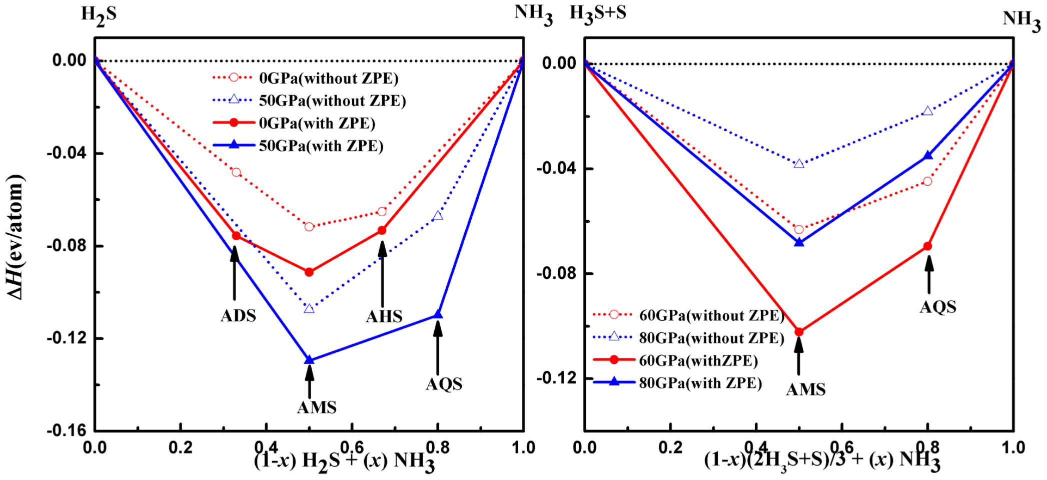

Supplementary Figure 10. Convex hulls of H_2S-NH_3 mixtures at selected pressures, without (dashed lines) and with (solid lines) ZPE corrections to ground state enthalpies.

Formation of NH3-H3S-S compounds

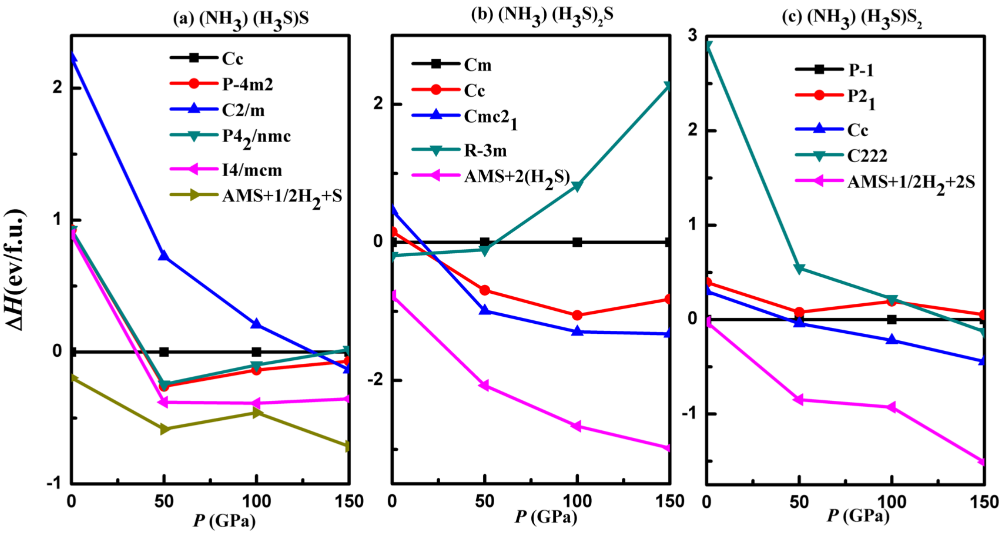

Supplementary Figure 11. Relative formation enthalpies for candidate NH_3-H_3S-S mixtures, relative to formation of H_2S-NH_3 mixtures.

Phase stability and properties of ATS under pressure

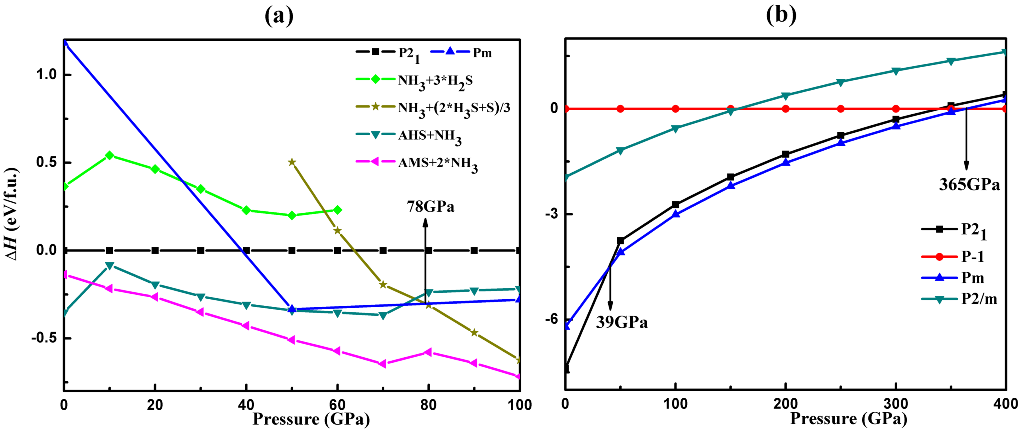

Supplementary Figure 12. The enthalpy differences per formula of ATS as a function of pressure.

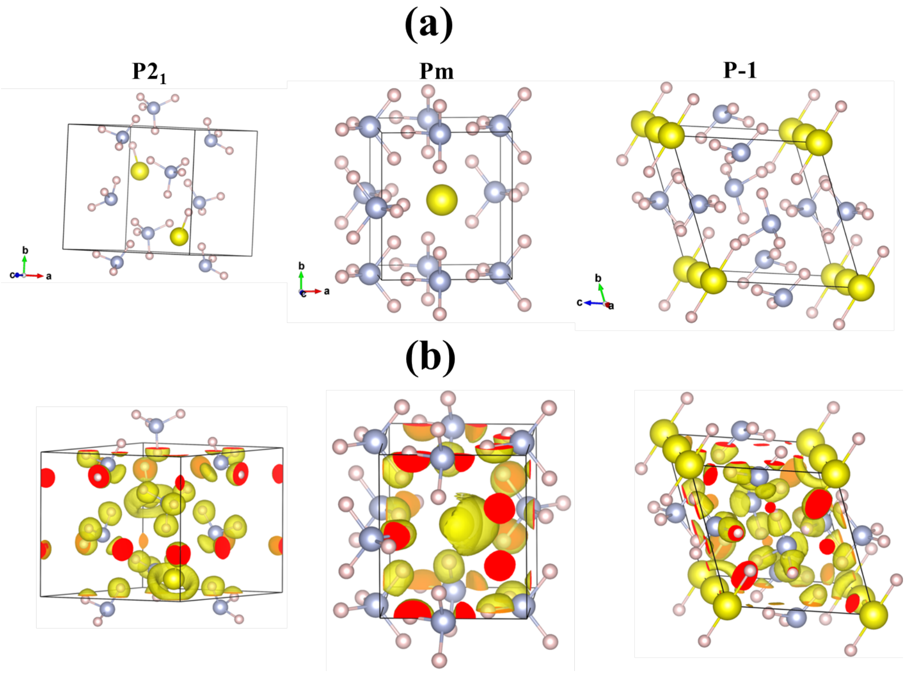

Supplementary Figure 13. The crystal structures (a) and ELF (b) of ATS.

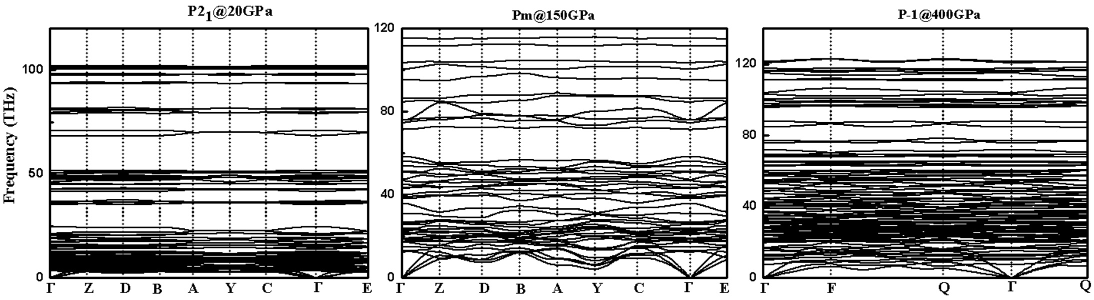

Supplementary Figure 14. Phonon dispersion curves of ATS.

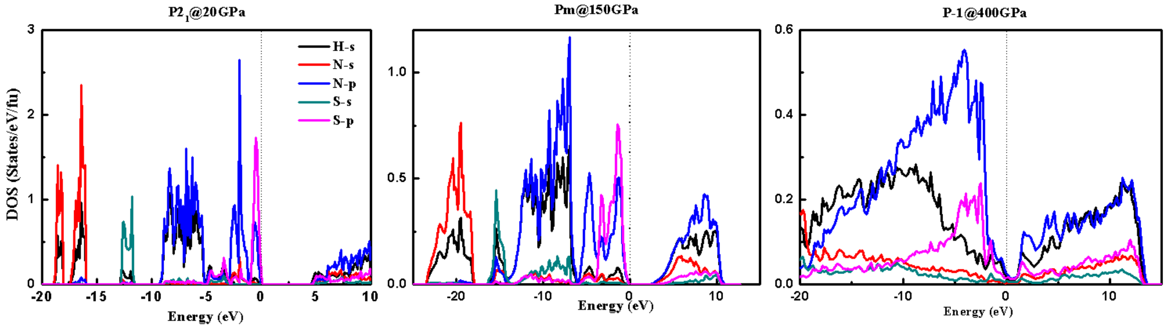

Supplementary Figure 15. The partial density of states of ATS under pressure.

Electron localization function (ELF)

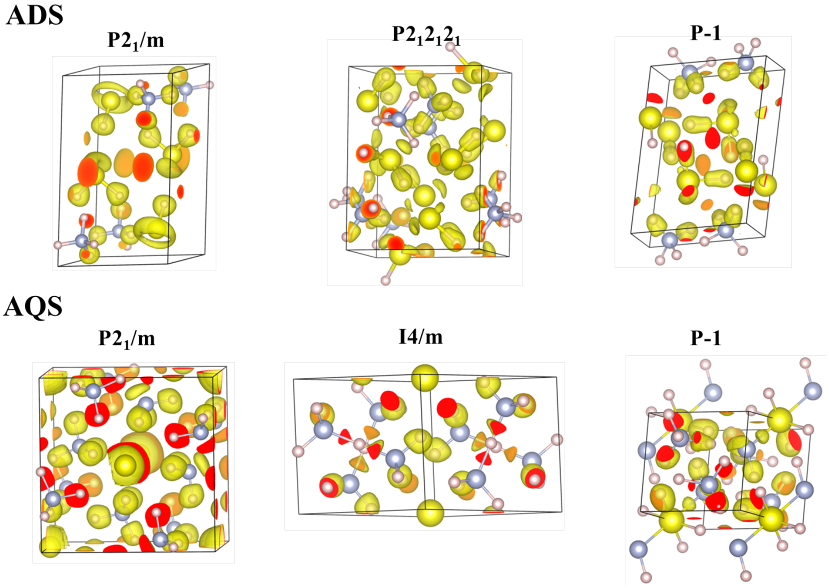

Supplementary Figure 16. ELF with an isosurface value of 0.85 of ADS and AQS.

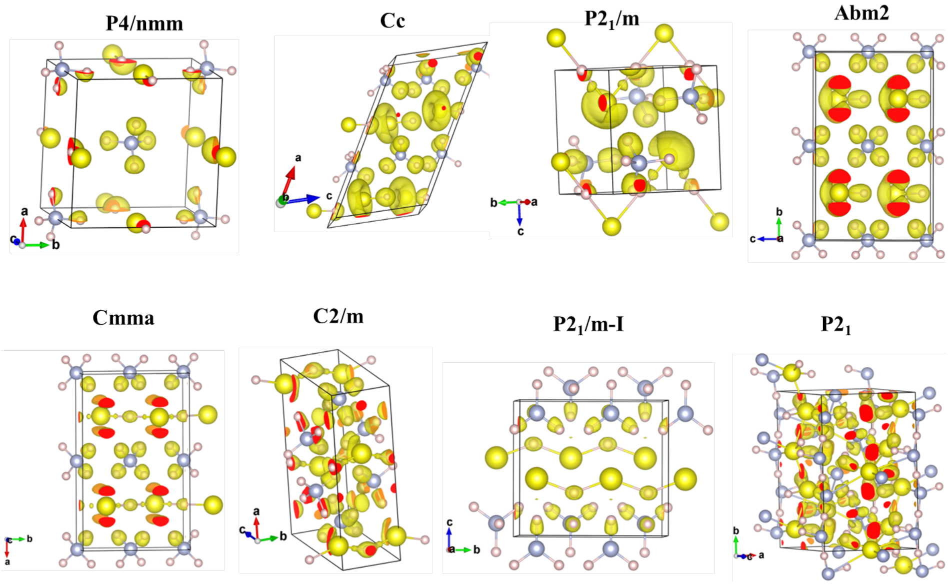

Supplementary Figure 17. ELF with an isosurface value of 0.85 of AMS.

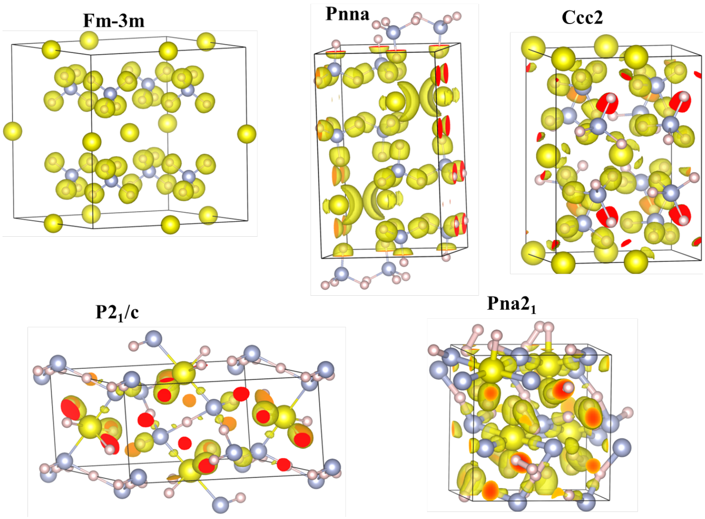

Supplementary Figure 18. ELF with an isosurface value of 0.85 of AHS.

Crystal structure representations

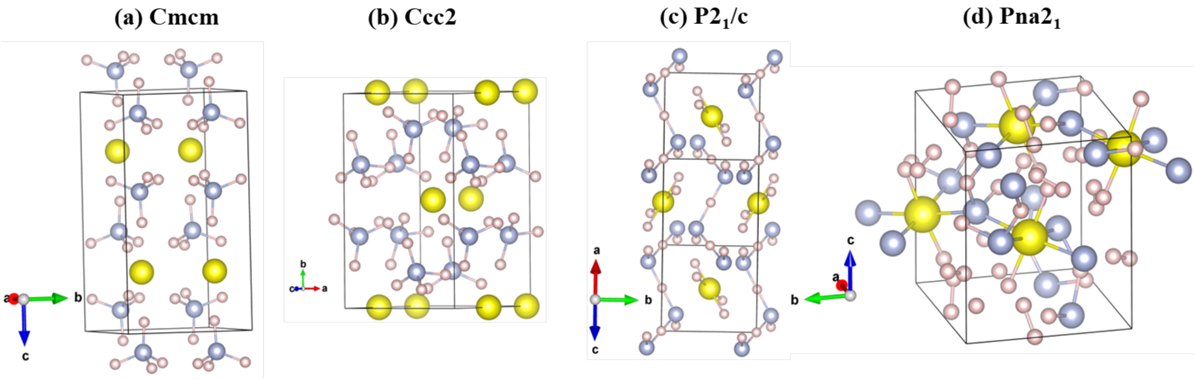

Supplementary Figure 19. The crystal structures of AHS.

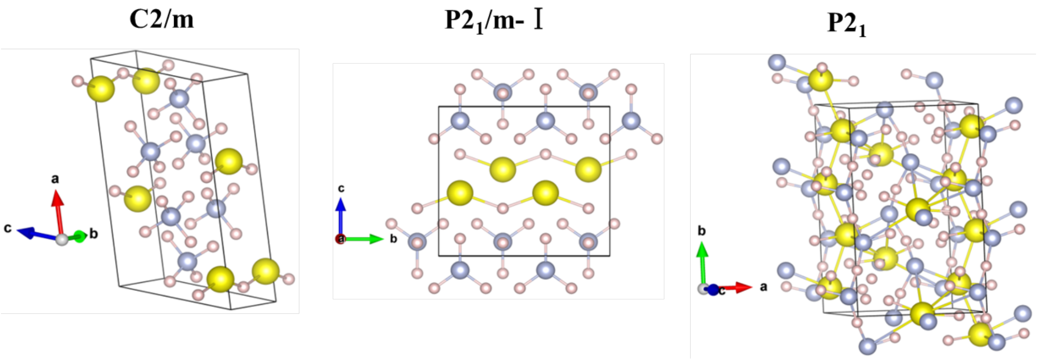

Supplementary Figure 20. The crystal structures of AMS.

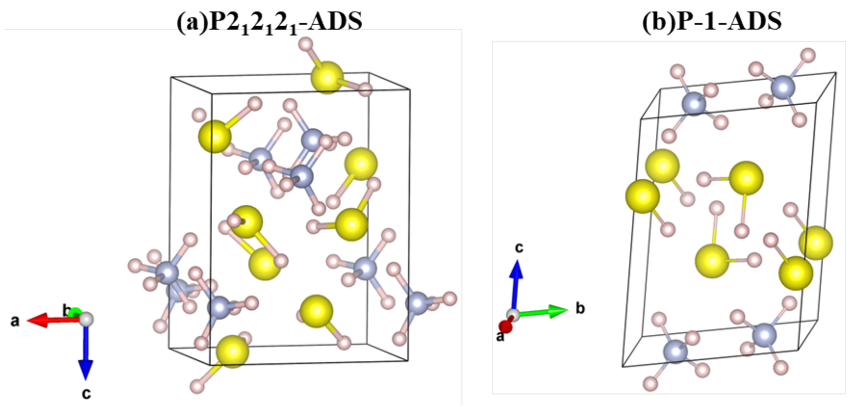

Supplementary Figure 21. The crystal structures of ADS.

Charge transfer and COHP in H_3S

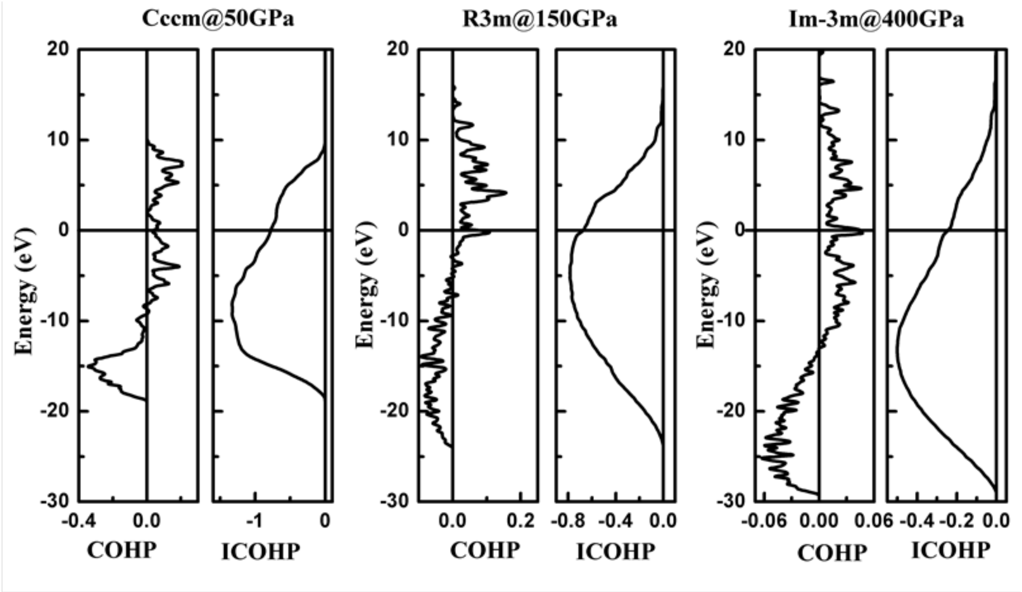

Supplementary Figure 22. The transferring charge of S atom (a) and (b) COHP and ICOHP in H_3S.

Partial densities of state (pDOS)

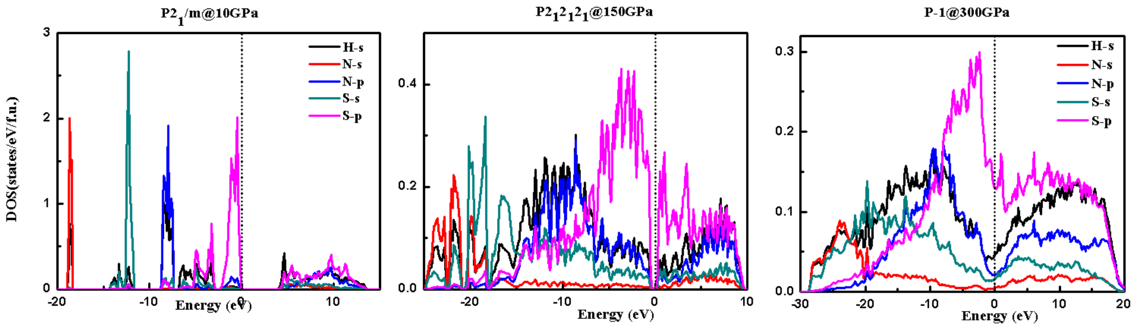

Supplementary Figure 23. The partial density of states of ADS under pressure.

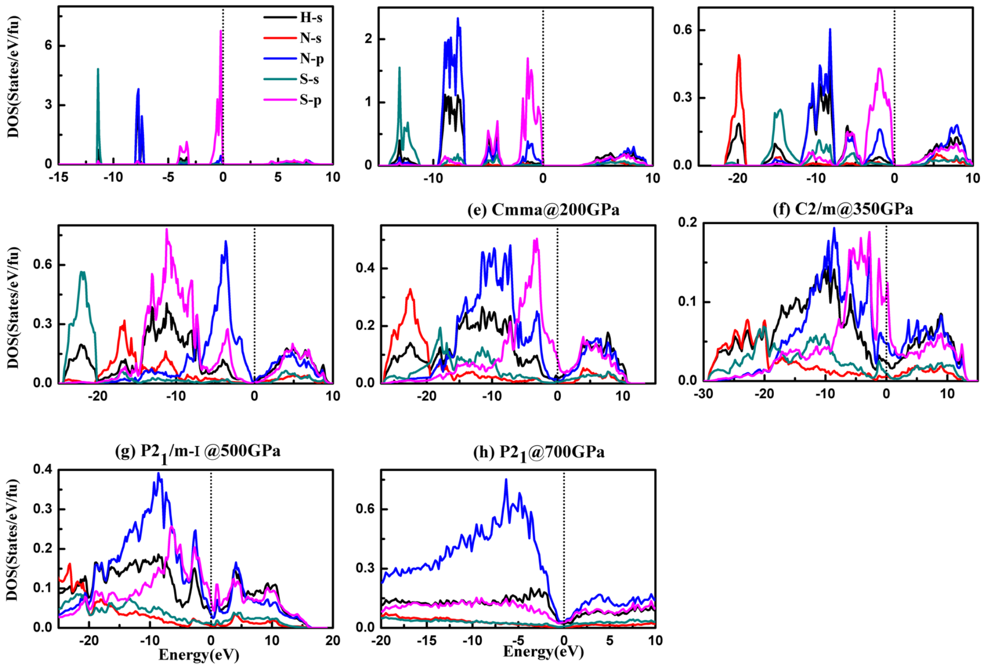

Supplementary Figure 24. The partial density of states (a-h) of AMS under pressure.

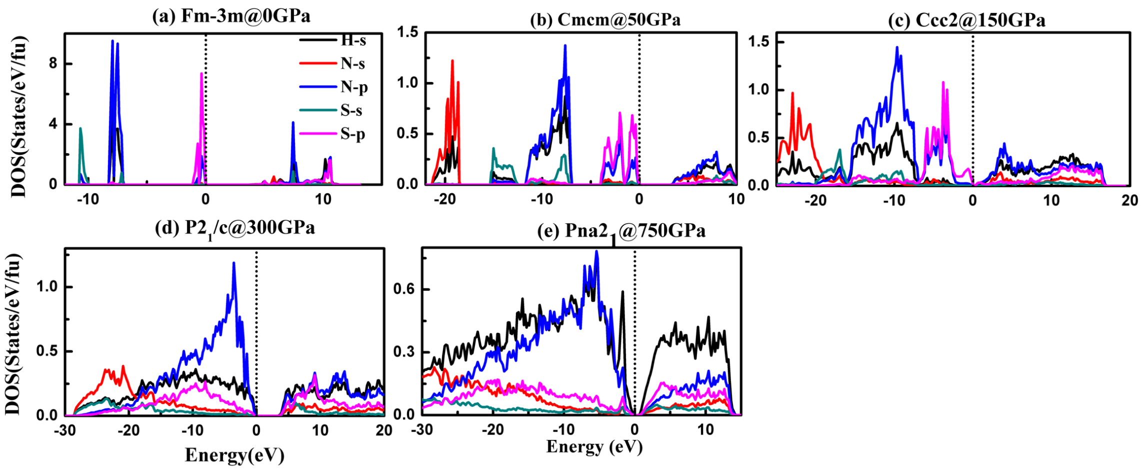

Supplementary Figure 25. The partial density of states (a-e) of AHS under pressure.

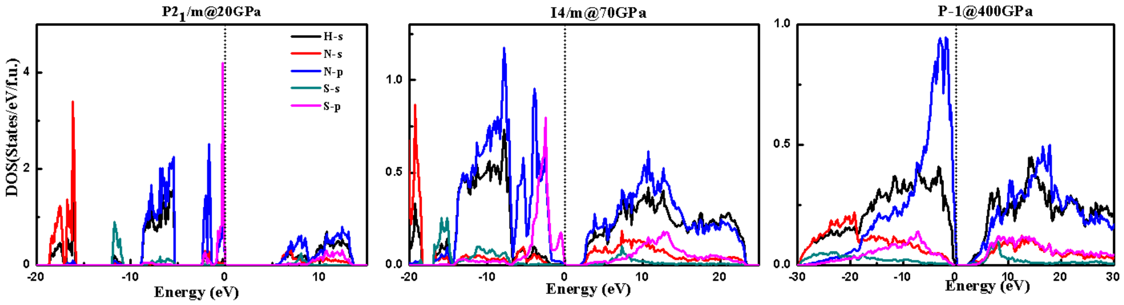

Supplementary Figure 26. The partial density of states of AQS under pressure.

Crystallographic Information

	Lattice constants (Å)	Atom	Atom	Positions	
		H (4f)	0.267	0.085	0.876
$P2_1/m$@10GPa	a =3.836	H (4f)	0.693	0.443	0.650
	b =5.178	H (2e)	0.122	0.750	0.046
	c =8.194	H (2e)	0.934	0.750	0.256
	$\alpha=\gamma=90^{o}$	H (2e)	0.610	0.250	0.287
	$\beta=77.9^{o}$	N (2e)	0.115	0.250	0.864
		S (2e)	0.578	0.750	0.836
		S (2e)	0.203	0.750	0.463
$P2_12_12_1$@150GPa		H (4a)	0.036	0.534	0.119
	a =4.637	H (4a)	0.452	0.806	0.687
	b =4.282	H (4a)	0.221	0.554	0.469
	c =7.703	H (4a)	0.902	0.799	0.998
	$\alpha=\beta=\gamma=90^{o}$	H (4a)	0.969	0.647	0.649
		H (4a)	0.216	0.439	0.772
		H (4a)	0.824	0.387	0.328
		N (4a)	0.609	0.823	0.781
		S (4a)	0.353	0.175	0.045
		S (4a)	0.116	0.772	0.876
P-1@300GPa		H (2i)	0.678	0.877	0.899
	a =2.346	H (2i)	0.773	0.616	0.133
	b =4.338	H (2i)	0.570	0.669	0.334
	c =6.041	H (2i)	0.777	0.146	0.895
	$\alpha=78.6\ ^{o}$	H (2i)	0.158	0.626	0.927
	$\beta=100.1^{o}$	H (2i)	0.384	0.836	0.503
	$\gamma=91.2^{o}$	H (2i)	0.029	0.376	0.497
		N (2i)	0.459	0.747	0.012
		S (2i)	0.279	0.398	0.313
		S (2i)	0.974	0.954	0.311

Supplementary Table 1. Structural information for predicted structures of ADS at relevant pressures.

	Lattice constants (Å)	Atom	Atom	Position

P4/nmm@0GPa	a =b=6.091	H (8i)	0.643	0.5	0.368
	c=4.454	H (2c)	0.5	0	0.082
	α=β=γ=90º	N (2b)	0.5	0.5	0.5
		S (2c)	0.5	0	0.779
Cc@20GPa	a =9.299	H (4a)	0.281	0.254	0.827
	b =4.610	H (4a)	0.437	0.076	0.803
	c =4.613	H (4a)	0.929	0.057	0.290
	α=γ=90º	H (4a)	0.609	0.545	0.178
	β=119.3º	H (4a)	0.285	0.743	0.964
		N (4a)	0.358	0.256	0.721
		S (4a)	0.609	0.234	0.470
$P2_1/m$@80GPa	a =3.604	H (4f)	0.165	0.547	0.735
	b =4.108	H (2e)	0.197	0.25	0.422
	c =4.099	H (2e)	0.129	0.25	0.015
	α=β=γ=90º	H (2b)	0.5	0	0
		N (2e)	0	0.25	0.238
		S (2e)	0.449	0.75	0.273
Abm2@100GPa	a =2.701	H (8d)	0.731	0.921	0.651
	b =8.074	H (8d)	0.269	0.074	0.931
	c =5.061	H (4c)	0.569	0.75	0.077
	α=β=γ=90º	S (4c)	0.125	0.75	0.795
		N (4b)	0.5	0.5	0.044
Cmca@200GPa	a =7.648	H (16o)	0.339	0.884	0.756
	b =4.668	H (4d)	0.5	0	0.5
	c =2.481	N (4b)	0.75	0.5	0.5
	α=β=γ=90º	S (4g)	0.5	0.75	0.129
C2/m@350GPa	a =7.139	H (8j)	0.553	0.292	0.616
	b =2.756	H (4i)	0.574	0	0.070
	c =3.868	H (4i)	0.271	0	0.696
	α=γ=90º	H (4i)	0.732	0	0.710
	β=74.2º	N (4i)	0.363	0	0.444
		S (4i)	0.872	0	0.939
$P2_1/m$- II @500GPa	a =2.420	H (4f)	0.762	0.552	0.783
	b =2.678	H (2e)	0.782	0.75	0.317
	c =4.853	H (2e)	0.212	0.75	0.885
	α=γ=90º	H (2e)	0.319	0.25	0.913
	β=89.4º	N (2e)	0.268	0.75	0.096

		S (2e)	0.741	0.25	0.421
$P2_1$@700GPa	a =4.319	H (2a)	0.772	0.727	0.692
	b =3.580	H (2a)	0.273	0.137	0.885
	c =3.917	H (2a)	0.987	0.943	0. 016
	α=γ=90º	H (2a)	0.461	0.185	0.531
	β=64.9º	H (2a)	0.653	0.997	0.603
		H (2a)	0.139	0.142	0.723
		H (2a)	0.154	0.892	0.156
		H (2a)	0.795	0.841	0.322
		H (2a)	0.662	0.274	0.221
		H (2a)	0.187	0.470	0.110
		N (2a)	0.105	0.701	0.847
		N (2a)	0.561	0.445	0.611
		S (2a)	0.081	0.762	0.482
		S (2a)	0.493	0.501	0.024

Supplementary Table 2. Structural information for predicted structures of AMS at relevant pressures.

	Lattice constants (Å)	Atom	Atom	Position	
Fm-3m@0GPa	a =b=c=6.574	H (32f)	0.669	0.332	0.331
	α=β=γ=90º	N (8c)	0.250	0.750	0.750
		S (4a)	0.5	0.5	0.5
Cmcm@80GPa	a =8.402	H (16h)	0.384	0.258	0.429
	b =5.081	H (8g)	0.893	0.044	0.250
	c =4.692	H (8g)	0.713	0.877	0.250
	α=β=γ=90º	N (8g)	0.838	0.857	0.250
		S (4c)	0.5	0.146	0.750
	a =4.068	H(8d)	0.491	0.697	0.378
Ccc2@150GPa	b =6.969	H(8d)	0.387	0.106	0.729
	c =5.099	H(8d)	0.646	0.914	0.421
	α=β=γ=90º	H(8d)	0.337	0.847	0.630
		N(8d)	0.509	0.833	0.289
		S(4a)	0	0	0.550
	a =3.378	H(4e)	0.801	0.864	0.768
	b =3.464	H(4e)	0.243	0.847	0.651
$P2_1/c$@400GPa	c =6.594	H(4e)	0.074	0.656	0.934
	α=γ=90º	H(2b)	0.5	0	0.5

	β=140.2º	H(2a)	0	0	0
		N(4e)	0.429	0.148	0.118
		S(2d)	0.5	0	0.5
	a =4.382	H(4a)	0.459	0.073	0.489
	b =3.875	H(4a)	0.369	0.114	0.629
$Pna2_1$@750GPa	c =4.575	H(4a)	0.787	0.562	0.533
	α=β=γ=90º	H(4a)	0.486	0.163	0.162
		H(4a)	0.874	0.869	0.155
		H(4a)	0.687	0.984	0.779
		H(4a)	0.088	0.388	0.726
		H(4a)	0.859	0.104	0.644
		N(4a)	0.128	0.248	0.344
		N(4a)	0.873	0.248	0.971
		S(4a)	0.372	0.503	0.398

Supplementary Table 3. Structural information for predicted structures of AHS at relevant pressures.

	Lattice constants (Å)	Atom	Atom Positions		
$P2_1/m$@20GPa	a=6.549	H (4f)	0.469	0.932	0.285
	b=4.623	H (4f)	0.026	0.927	0.205
	c=6.520	H (4f)	0.207	0.932	0.472
	α=γ=90º	H (4f)	0.287	0.927	0.025
	β=89.9º	H (2e)	0.287	0.25	0.748
		H (2e)	0.757	0.25	0.283
		H (2e)	0.412	0.25	0.522
		H (2e)	0.091	0.25	0.979
		H (2e)	0.983	0.25	0.409
		H (2e)	0.523	0.25	0.084
		N (2e)	0.935	0.75	0.177
		N (2e)	0.174	0.75	0.561
		N (2e)	0.558	0.75	0.936
		N (2e)	0.321	0.75	0.936
		S (2e)	0.253	0.25	0.261
I4/m@70GPa	a =b=5.968	H (16i)	0.971	0.215	0.804
	c=4.178	H (4c)	0	0.5	0
	α=β=γ=90º	H (8h)	0.742	0.179	0.5
		N (8h)	0.571	0.192	0.5
		S (2b)	0	0	0.5

P-1@400GPa	a=3.469	H (2i)	0.486	0.301	0.688
	b=3.561	H (2i)	0.808	0.421	0.525
	c=3.469	H (2i)	0.235	0.970	0.052
	α= 104.1º	H (2i)	0.136	0.521	0.898
	β=104.0º	H (2i)	0.657	0.116	0.424
	γ=94.5º	H (2i)	0.894	0.797	0.127
		H (2i)	0.538	0.707	0.836
		N (2i)	0.228	0.311	0.375
		N (2i)	0.646	0.267	0.967
		S (1b)	0	0	0.5

Supplementary Table 4. Structural information for predicted structures of AQS at relevant pressures.

	Lattice constants (Å)	Atom	Atom Position		
$P2_1$@20GPa	a=5.674	H (2a)	0.482	0.236	0.309
	b=5.576	H (2a)	0.307	0.238	0.492
	c=5.671	H (2a)	0.556	0.562	0.179
	α=γ=90º	H (2a)	0.182	0.565	0.562
	β=114.6º	H (2a)	0.765	0.967	0.149
		H (2a)	0.152	0.972	0.765
		H (2a)	0.016	0.340	0.601
		H (2a)	0.604	0.337	0.018
		H (2a)	0.173	0.154	0.174
		H (2a)	0.380	0.971	0.382
		H (2a)	0.957	0.340	0.958
		N (2a)	0.802	0.912	0.335
		N (2a)	0.334	0.912	0.335
		N (2a)	0.658	0.650	0.658
		S (2a)	0.171	0.640	0.172
Pm@150GPa	a =3.352	H (2c)	0.425	0.213	0.274
	b =3.745	H (2c)	0.774	0.712	0.049
	c =3.756	H (2c)	0.139	0.779	0.604
	α=γ=90.0º	H (1a)	0.176	0	0.959
	β=89.9º	H (1a)	0.707	0	0.759
		H (1a)	0.841	0	0.336
		H (1b)	0.088	0.5	0.333
		H (1b)	0.185	0.5	0.902
		N (1a)	0.432	0	0.089
		N (1a)	0.944	0	0.583
		N (1b)	0.952	0.5	0.090

		S (1b)	0.510	0.5	0.597
P-1@400GPa	a =3.759	H (2i)	0.030	0.471	0.213
	b =4.404	H (2i)	0.237	0.685	0.907
	c =4.316	H (2i)	0.260	0.379	0.542
	α=68.9º	H (2i)	0.023	0.736	0.516
	β=90.2º	H (2i)	0.371	0.741	0.332
	γ=89.2º	H (2i)	0.144	0.064	0.291
		H (2i)	0.316	0.309	0.013
		H (2i)	0.934	0.722	0.217
		H (2i)	0.517	0.518	0.759
		H (2i)	0.517	0.819	0.567
		H (2i)	0.761	0.081	0.311
		N (2i)	0.241	0.106	0.487
		N (2i)	0.255	0.487	0.098
		S (1a)	0	0	0
		S (1d)	0.5	0	0

Supplementary Table 5. Structural information for predicted structures of ATS at relevant pressures.

Bader charge transfer

ADS				ATS			
Phase	Pressure	Charges (e)		Phase	Pressure	Charges (e)	
$P2_1/m$	10GPa	H1(8)	+0.443	$P2_1$	20GPa	H1(22)	+0.422
		H2(6)	+0.049			N(6)	-1.184
		N(2)	-1.137			S (2)	-0.650
		S1(2)	-0.613				
		S2(2)	-0.189	Pm	100GPa	H(11)	+0.442
						N(3)	-1.242
$P2_12_12_1$	150GPa	H1(4)	-0.016			S (1)	-0.928
		H2(16)	+0.419				
		H3(8)	-0.018	P-1	400GPa	H1(20)	+0.416
		N(4)	-1.195			H2(2)	-0.216
		S1(4)	-0.146			N(6)	-1.271
		S2(4)	-0.313			S1(1)	-0.407
						S2 (1)	+0.158
P-1	300GPa	H1(8)	+0.381				
		H2(4)	+0.031				
		H3(2)	-0.012				
		N(2)	-1.189				
		S1(2)	-0.091				
		S2(2)	-0.289				

Supplementary Table 6. Bader charges in ADS and ATS.

AMS				AHS			
Phase	Pressure		Charges (e)	Phase	Pressure		Charge(e)
P4/nmm	0GPa	H1(8)	+0.446	Fm-3m	0GPa	H(8)	+0.452
		H2(2)	-0.0587			N(2)	-1.176
		N(2)	-1.103			S	-1.230
		S(2)	-0.623				
				Cmcm	50GPa	H(32)	+0.427
Cc	20GPa	H1(8)	+0.448			N(8)	-1.200
		H2(2)	+0.082			S(4)	-1.040
		N(2)	-1.179				
		S(2)	-0.694	Ccc2	160GPa	H(16)	+0.412
						N(4)	-1.204
$P2_1/m$	70GPa	H1(8)	+0.432			S(2)	-0.886
		H2(2)	+0.159				
		N(2)	-1.184	$P2_1/c$	300GPa	H1(8)	+0.466

		S(2)	-0.694			H2(8)	-0.241
						N(4)	-1.527
Abm2	100GPa	H1(8)	+0.418			S(2)	+2.211
		H2(2)	+0.069				
		N(2)	-1.181	$Pna2_1$	750GPa	H1(8)	+0.438
		S(2)	-0.559			H2(8)	+0.174
						H3(4)	-0.086
Cmma	150GPa	H	+0.331			H4(12)	-0.223
		N	-1.098			N(8)	-1.847
		S	-0.559			S(4)	+3.172
C2/m	350GPa	H1(8)	+0.411				
		H2(2)	+0.124				
		N(2)	-1.155				
		S(2)	-0.311				
$P2_1/m$- Ⅰ	500GPa	H1(10)	+0.325				
		N(2)	-1.183				
		S(2)	-0.168				
$P2_1$	700GPa	H1(6)	+0.442				
		H2(2)	+0.133				
		H3(8)	-0.221				
		H4(4)	-0.056				
		N(4)	-1.649				
		S(4)	+1.417				

Supplementary Table 7. Bader charges in AMS and AHS.

Phase	Pressure	Charges (e)	
$P2_1/m$	20GPa	H(28)	+0.431
		N (8)	-1.208
		S (2)	-1.124
I4/m	70GPa	H (14)	+0.425
		N (4)	-1.221
		S(1)	-1.010
P-1	400GPa	H1(10)	+0.442
		H2(6)	-0.261
		N1(2)	-1.642
		N2(2)	-1.273
		S(1)	+2.454

Supplementary Table 8. Bader charges in AQS under pressure. + and – represent lost and gained electrons, respectively.

Phase	Pressure	Bond critical point (BCP)	Charge ρ_{BCP} (e)	Laplacian $\nabla^2\rho_{BCP}$ (e/Å^2)
I4/m	70GPa	N-H(16i)	0.339	-1.616
		N-H(8h)	0.341	-2.292
		N-H(4c)	0.192	-0.489
		S…H(16i)	0.071	0.062
		S…H(8c)	0.063	0.086
P-1	400GPa	N-H (NH_4^+)	0.413, 0.412	-2.601, -2.117
		N-H (NH_4^+)	0.336, 0.335	-1.475, -1.493
		N-H (NH^-)	0.407	-2.041
		S-N	0.296	-0.290
		S-H	0.293, 0.287	-1.041, -0.982

Supplementary Table 9. Bader topology analysis in AQS under pressure.

Superconducting temperatures and relevant parameters

Phase	Pressure	λ	ω_{log} (K)	T_c (K)
AMS				
C2/m	350GPa	0.681	928	30.5
P21/m- I	500GPa	0.590	1269	28.3
	600GPa	0.570	1413	28.1
$P2_1$	700GPa	0.405	1261	5.6
P-1-ADS	300GPa	1.005	667	46.4
Ccc2-AHS	180GPa	0.395	1029	4.0

Supplementary Table 10. Calculated EPC Parameter (λ), Logarithmic Average Phonon Frequency (ω_{log}) and superconducting Temperature (T_c) for stable compounds of NH_3-H_2S system at given pressures.

Supplementary References

1. Perdew, J.P., Burke, K. & Ernzerhof, M. Generalized gradient approximation made simple. *Phys. Rev. Lett.* **77**: 3865 (1996).

2. Kresse, G. & Furthmüller, J. Efficient iterative schemes for ab initio total-energy calculations using a plane-wave basis set. *Phys. Rev.* B **54**: 11169 (1999).

3. Parlinski, K., Li, Z.Q. & Kawazoe, Y., First-Principles Determination of the Soft Mode in Cubic ZrO_2. *Phys. Rev. Lett.* **78**: 4063 (1997).

4. Togo, A., Oba, F. & Tanaka, I. First-principles calculations of the ferroelastic transition between rutile-type and $CaCl_2$-type SiO_2 at high pressures. *Phys. Rev.* B **78**: 134106 (2008).

5. Bader, R.F.W. Atoms in Molecules: A Quantum Theory (Oxford University Press, Oxford, UK) (1994).

6. Giannozzi, P., et.al. QUANTUM ESPRESSO: a modular and open-source software project for quantum simulations of materials. *J. Phys.: Condens. Matter* **21**: 395502 (2009).

7. Mcmillan, W.L. Transition temperature of strong-coupled superconductors. *Phys. Rev.* **167**: 331-344 (1968).

8. Robinson, V.N., Marqués, M., Wang, Y.C., Ma, Y.M. & Hermann, A. Novel phases in ammonia-water mixtures under pressure. *J. Chem. Phys.* **149**: 234501 (2018).

9. Blaha, P., Schwarz, K., Tran, F., Laskowski, R., Madsen, G.K.H. & L.D. Marks, J. Chem. Phys. **152**: 074101 (2020)
